# Anaerobic hydrocarbon and fatty acid metabolism by syntrophic bacteria and their impact on carbon steel corrosion

**DOI:** 10.3389/fmicb.2014.00114

**Published:** 2014-04-01

**Authors:** Christopher N. Lyles, Huynh M. Le, William Howard Beasley, Michael J. McInerney, Joseph M. Suflita

**Affiliations:** ^1^Department of Microbiology and Plant Biology, Institute for Energy and the Environment, and the OU Biocorrosion Center, University of OklahomaNorman, OK, USA; ^2^Howard Live Oak, LLCNorman, OK, USA

**Keywords:** syntrophy, biocorrosion, microbiologically influenced corrosion (MIC), hydrocarbon degradation, fatty acid oxidation

## Abstract

The microbial metabolism of hydrocarbons is increasingly associated with the corrosion of carbon steel in sulfate-rich marine waters. However, how such transformations influence metal biocorrosion in the absence of an electron acceptor is not fully recognized. We grew a marine alkane-utilizing, sulfate-reducing bacterium, *Desulfoglaeba alkanexedens*, with either sulfate or *Methanospirillum hungatei* as electron acceptors, and tested the ability of the cultures to catalyze metal corrosion. Axenically, *D. alkanexedens* had a higher instantaneous corrosion rate and produced more pits in carbon steel coupons than when the same organism was grown in syntrophic co-culture with the methanogen. Since anaerobic hydrocarbon biodegradation pathways converge on fatty acid intermediates, the corrosive ability of a known fatty acid-oxidizing syntrophic bacterium, *Syntrophus aciditrophicus* was compared when grown in pure culture or in co-culture with a H_2_-utilizing sulfate-reducing bacterium (*Desulfovibrio sp*., strain G11) or a methanogen (*M*. *hungatei*). The instantaneous corrosion rates in the cultures were not substantially different, but the syntrophic, sulfate-reducing co-culture produced more pits in coupons than other combinations of microorganisms. Lactate-grown cultures of strain G11 had higher instantaneous corrosion rates and coupon pitting compared to the same organism cultured with hydrogen as an electron donor. Thus, if sulfate is available as an electron acceptor, the same microbial assemblages produce sulfide and low molecular weight organic acids that exacerbated biocorrosion. Despite these trends, a surprisingly high degree of variation was encountered with the corrosion assessments. Differences in biomass, initial substrate concentration, rates of microbial activity or the degree of end product formation did not account for the variations. We are forced to ascribe such differences to the metallurgical properties of the coupons.

## Introduction

Anaerobic hydrocarbon biodegradation is metabolically coupled with the consumption of a variety of terminal electron acceptors (for recent reviews see, Callaghan, 2013; Heider and Schühle, 2013). It is essential to understand the underlying geochemical settings for such bioconversions in order to reliably assess the attendant environmental consequences. Arguably, the greatest impact of this metabolism is manifest in sulfate-laden environments where petroleum metabolism may be coupled with sulfide production. Aside from the destruction of the hydrocarbons *per se*, the formation of sulfide has several important consequences including health and safety concerns (Reiffenstein et al., 1992), reservoir souring (Jenneman et al., 1986; McInerney et al., 1996; Nemati et al., 2001; Hubert and Voordouw, 2007), and metal biocorrosion (Hamilton, 1985). Not surprisingly, these problems are most acute in marine environments where sulfate-reducing bacterial communities thrive in petroleum deposits, hydrocarbon seeps, petroleum hydrothermal sediments, methane hydrates, oil field equipment, and in hydrocarbon-contaminated sediments (Teske, 2010). Moreover, in offshore and near coastal drilling operations where seawater is injected into petroleum reservoirs to maintain oil field formation pressures, efforts are regularly made to remove sulfate from seawater in order to control the deleterious consequences of scale deposits as well as to minimize microbial sulfate reduction, reservoir souring, and sulfide-induced metal corrosion (Bader, 2007). When exogenous electron acceptors are limiting, anaerobic hydrocarbon mineralization can still proceed and result in the formation of methane (Dolfing et al., 2008; Gieg et al., 2008; Jones et al., 2008; Heider and Schühle, 2013). In fact, methanogenic enrichments and isolates are regularly obtained from hydrocarbon-rich marine ecosystems (Teske, 2010), even though these organisms are not known to directly utilize petroleum components. Rather, acetoclastic and hydrogenotrophic methanogens catalyze the terminal mineralization steps as members of thermodynamically-based hydrocarbonoclastic syntrophic microbial assemblages of varying complexity (Dolfing et al., 2008; Gieg et al., 2008; Jones et al., 2008). The consequences of methanogenic hydrocarbon metabolism minimally include the overall diminution in the quality of petroleum reserves (Head et al., 2010), and the formation of methane a powerful greenhouse gas (Blake and Rowland, 1988). The other end product of this bioconversion, carbon dioxide, can potentially alter the *in situ* mineralization pathways in petroliferous reservoirs. If carbon dioxide is in a high enough concentration, acetoclastic methanogenesis may become a dominant process and increase the rate of methane production and sequestration of carbon dioxide (Mayumi et al., 2013).

Thus, the complete mineralization of hydrocarbons under anaerobic conditions, like the biodegradation of other complex forms of organic matter, can be initiated or accomplished by specialized microbial isolates that can couple this metabolism to the consumption of exogenous electron acceptors or enter into complex syntrophic partnerships. Such bioconversions often result in the transient formation of fatty acid metabolites (e.g., acetate, propionate, butyrate, and benzoate) that have been postulated as intermediates of anaerobic hydrocarbon metabolism under both methanogenic and sulfate-reducing conditions (Cozzarelli et al., 1994; Van Stempvoort et al., 2007, 2009; Struchtemeyer et al., 2011). In syntrophic partnerships, it is well known that non-methanogens, such as hydrogen/formate-utilizing, sulfate-reducing bacteria (SRB), can fulfill the role of the terminal electron-accepting microorganism (Hopkins et al., 1995; Warikoo et al., 1996; Jackson et al., 1999). Conversely, a syntrophic association with methanogens can also be realized even in environments with high sulfate concentrations (Struchtemeyer et al., 2011).

The role of SRB and methanogens as agents of metal corrosion, when grown as pure cultures or as members of syntrophic consortia, is not fully appreciated. These organisms have been implicated in the corrosion of metal via direct electron transfer from zero-valent iron under electron donor-limited conditions (Dinh et al., 2004; Uchiyama et al., 2010; Enning et al., 2012; Enning and Garrelfs, 2014). Additionally, when electron donors are sufficient, the inorganic and organic compounds produced during metabolism (e.g., hydrogen, fatty acids, carbon dioxide, and sulfides) have frequently been associated with metal biocorrosion (King and Miller, 1971; King and Wakerley, 1972; King et al., 1973a,b; Crolet et al., 1999; Hedges and McVeigh, 1999; Garsany et al., 2003; Kermani and Morshed, 2003; Suflita et al., 2008).

Given the diverse modes of existence for the requisite microorganisms, we used defined incubations of both pure and co-culture syntrophic bacteria to examine their impact on carbon steel biocorrosion. More specifically, we evaluated a known hydrocarbon-degrading bacterium, *Desulfoglaeba alkanexedens* strain ALDC (Davidova et al., 2006), grown with sulfate or syntrophically in co-culture with a methanogen as the electron acceptor. *D. alkanexedens* strain ALDC is a marine alkane-degrading, sulfate-reducing bacterium, that can completely oxidize C_6_–C_12_ *n*-alkanes via the fumarate addition pathway. The bacterium can also syntrophically degrade the same suite of aliphatic hydrocarbons in a sulfate-free medium when co-cultured with the hydrogen/formate-consuming methanogen, *Methanospirillum hungatei* strain JF-1 (Ferry et al., 1974). We also evaluated biocorrosion with a known fatty acid-oxidizing syntrophic bacterium, *Syntrophus aciditrophicus* strain SB (Hopkins et al., 1995; Jackson et al., 1999), grown as a pure culture or with either a hydrogen/formate-utilizing methanogen or SRB as the electron-accepting microorganism. *S. aciditrophicus* strain SB, metabolizes various saturated and unsaturated fatty acids, methyl esters of butyrate and hexanoate, benzoate, and alicyclic acids when grown in co-culture with a hydrogen/formate-consuming microorganism (Mouttaki et al., 2007) including the methanogen *M. hungatei* strain JF-1 or the SRB *Desulfovibrio sp*. strain G11 (McInerney et al., 1979). Its use of a primary sodium pump to create a chemostatic potential and to synthesize ATP using a sodium gradient mimics the bioenergetic scheme of many marine microorganisms (McInerney et al., 2007). Similarly, *M. hungatei* strain JF-1 also has the capacity to produce and use sodium gradients (Anderson et al., 2009). *S. aciditrophicus* strain SB can also be grown as a pure culture on crotonate whereupon it produces acetate, cyclohexane carboxylate, and benzoate as metabolic end products (Mouttaki et al., 2007). Lastly, all pure cultures were individually evaluated for their ability to corrode metal coupons. Our results suggest that the corrosion of carbon steel was generally more evident when anaerobic microbial metabolism was linked to sulfate reduction rather than methanogenesis. However, a greater than expected standard deviation in corrosivity was measured in replicate incubations. After controlling and monitoring the biological and chemical characteristics of the incubations, we are forced to attribute the variability to presumed compositional differences in the metal used for the construction of the coupons.

## Materials and methods

### Electrochemical cell construction

**Figure 1:**
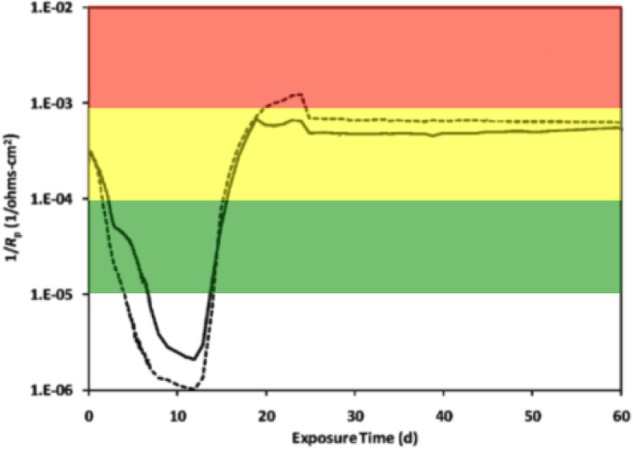
**An instantaneous corrosion rate (1/*R_p_*) curve adapted from Aktas et al. (2010)**. The basic shape of the curve represents the corrosion of C1020 metal over time. 1/*R*_*p*_ = (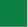) 10^−5^ ohms^−1^ cm^−2^, <0.0254 mmpy; (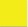) 10^−4^ ohms^−1^ cm^−2^, 0.0254–0.127 mmpy; (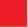) 10^−3^ ohms^−1^ cm^−2^, 0.127–0.508 mmpy.

Electrochemical cells were made from culture bottles (100 ml; Figure S1) fitted with rubber stoppers that were modified to hold a graphite counter electrode and a Luggin probe filled with 1 M potassium chloride (KCl) solution containing a saturated calomel reference electrode (Gamry Instruments, Warminster, PA; Figure S2). The working electrode was a 1020 carbon steel coupon (Alabama Specialty Products, Munford, AL) with dimensions of 0.95 cm diameter × 1.27 cm and a surface area of 4.5 cm^2^. The metal was prepared using the specifications detailed in ASTM Standard A576-90b (2006), and consisted of the elemental components listed in Table S1 according to the manufacturer (above). A wire was attached to the coupon using rosin core solder and the entire assembly was sealed with epoxy resin (Figure S3). The working electrode assembly was then washed with acetone and methanol, before being sterilized by immersion for 30 min in a 70% ethanol bath. The ethanol was evaporated with an open flame, and the coupon was dried under nitrogen and handled aseptically. Sterile electrochemical probes (Figure S2) were placed in the incubations (Figure S4) while inside a well-working anaerobic chamber (N_2_:H_2_ 95:5%), and linear polarization resistance (LPR) curves were recorded every 5 min for a 30 min period using computer controlled potentiostats (model 600, Gamry Instruments, Warminster, PA). The potential was swept ± 10 mV above and below the corrosion potential (*E*_*corr*_) at a rate of 0.125 mV/s. Tafel slope regions were used to extrapolate resistance polarization (*R_p_*) values within ± 5 mV of the *E*_*corr*_. The instantaneous corrosion rate is derived by taking the inverse of the resistance polarization (1/*R_p_*; ohms^−1^ cm^−2^). The LPR measurement was taken intermittently based on the metabolic activity measured within anaerobic incubations (below). Thus, the 1/*R_p_* curves reflect various points along the curve shown in Figure 1. A 1/*R_p_* value for each time point was selected based on the mean distribution of six LPR curves within the 30 min measurement period. Finally, instantaneous corrosion rates were generalized as low [<0.0254 millimeter per year (mmpy)], moderate (0.0254–0.127 mmpy), or high (0.127–0.508 mmpy) based on the 1/*R_p_* logarithmic value (Figure 1).

### Cultivation of the hydrocarbon-degrading bacterium

The mineral medium was prepared as previously described (McInerney et al., 1979), except rumen fluid and sulfate were omitted. The medium was amended with 10 ml/L of a trace metal and vitamin solution (Tanner, 2002), 0.0001% solution of resazurin as a redox indicator, sodium bicarbonate (24 mM), and 10 ml/L of a 2.5% solution of cysteine sulfide used as a reductant. The medium was dispensed into the electrochemical cells (50 ml) and inoculated with 20% transfers for the *D. alkanexedens* strain ALDC or *M. hungatei* strain JF-1 pure culture incubations or 10% transfers of each microorganism for the co-culture incubations. The mineral medium was the same for all axenic cultures or co-cultures, but the substrate, sulfate concentration, and headspace gas composition were adjusted for different incubation conditions (Table 1). The headspace for the axenic cultures of *M. hungatei* strain JF-1 was monitored and exchanged every 14 days to resupply H_2_/CO_2_ to support the hydrogenotrophic growth of the methanogen. Strict anaerobic conditions were maintained throughout the 44 days incubation period. Triplicate cultures were incubated at 32°C and monitored for corrosion with the potentiostats every 26 days after moving the cultures inside the anaerobic chamber. After each LPR measurement, the culture bottles were resealed and the headspace exchanged to the appropriate atmosphere.

**Table 1:** **Substrate, sulfate, and headspace amendments for hydrocarbon degrading incubations**.

**Axenic or co-culture incubations**	**Substrate**	**Sulfate (mM)**	**Headspace atmosphere**
*D. alkanexedens* ALDC	1 ml *n*-decane	20	138 kPa N_2_/CO_2_ (80:20)
*M. hungatei* JF-1	H_2_/CO_2_ (138 kPa in ~40 mls of headspace)	–	138 kPa H_2_/CO_2_ (5:95)
*D. alkanexedens* ALDC + *M. hungatei* JF-1	1 ml *n*-decane	–	138 kPa N_2_/CO_2_(80:20)
Uninoculated medium control	1 ml *n*-decane	20	138 kPa H_2_/CO_2_(80:20)

*“–”, No amendment*.

### Cultivation of the fatty acid-oxidizing syntroph

A basal medium was prepared as previously described (Tanner, 2002), and was amended with 10 ml/L trace metal and vitamin solution (Tanner, 2002), a 0.0001% solution of resazurin as a redox indicator, sodium bicarbonate (24 mM), and 20 ml/L of a 2.5% solution of cysteine sulfide used as a reductant. The medium was dispensed into the electrochemical cells (50 ml) and inoculated with 20% transfers of *S. aciditrophicus* strain SB or *Desulfovibrio sp*. strain G11 pure cultures, or 10% transfers of each of the microorganisms for the syntrophic co-culture incubations. The basal medium was the same for all cultures and co-cultures, but the substrate, sulfate concentration, and headspace atmosphere were adjusted depending on the desired incubation condition (Table 2). The headspace in autotrophically grown *Desulfovibrio sp*. strain G11 incubations was exchanged daily to resupply H_2_/CO_2_ to support the growth of this microorganism. Cultures were incubated in triplicate while medium controls were in duplicate. Incubations were held at 32°C and monitored electrochemically for the instantaneous corrosion rate as described above, every 7 days (experiment 1) or 28 days (experiment 2) after placing the cultures inside the anaerobic chamber. After each LPR measurement, the culture bottles were resealed and the headspace exchanged to the original atmosphere.

**Table 2:** **Substrate, sulfate, and headspace amendments for fatty acid oxidizing incubations**.

**Axenic or co-culture incubations**	**Substrate**	**Sulfate (mM)**	**Headspace atmosphere**
*S. aciditrophicus* SB	20 mM crotonate	–	138 kPa N_2_/CO_2_ (80:20)
*S. aciditrophicus* SB + *Desulfovibrio sp*. G11	20 mM crotonate	20	138 kPa N_2_/CO_2_ (80:20)
*S. aciditrophicus* SB + *M. hungatei* JF-1	20 mM crotonate	–	138 kPa N_2_/CO_2_ (80:20)
*Desulfovibrio sp*. G11 (heterotrophic)	20 mM lactate	20	138 kPa N_2_/CO_2_ (80:20)
*Desulfovibrio sp*. G11 (autotrophic)	H_2_/CO_2_ (138 kPa in ~40 mls of headspace)	20	138 kPa H_2_/CO_2_ (5:95)
*Desulfovibrio sp*. G11 (autotrophic)	H_2_ from metal surface	20	138 kPa N_2_/CO_2_ (80:20)
Uninoculated medium control	20 mM crotonate	–	138 kPa N_2_/CO_2_ (80:20)
Uninoculated medium control	20 mM lactate	20	138 kPa N_2_/CO_2_ (80:20)
Uninoculated medium control	–	20	138 kPa H_2_/CO_2_ or 138 kPa N_2_/CO_2_

*“–”, No amendment*.

### Spent medium experiments

The corrositivity of the basal medium (Tanner, 2002) was assessed after amendment with various fatty acids with concentrations of crotonate and lactate ranging from 0 to 20 mM, as well as acetate ranging from 0 to 40 mM. These compounds were exogenously amended to mimic the concentration of substrates and end products produced by a crotonate grown co-culture of *S. aciditrophicus* strain SB and *Desulfovibrio sp*. strain G11 and lactate metabolism by a pure culture of *Desulfovibrio sp*. strain G11. Acetate was amended at a ratio of 2:1 for crotonate metabolism and 1:1 for lactate metabolism. To represent sulfide production within the uninocultated incubations, sodium sulfide was amended at 10 and 20 mM concentrations. The pH for all incubations was ~7.0. The incubation conditions are described in Table 3. Duplicate uninoculated incubations were held at 32°C and monitored electrochemically every 17 days inside the anaerobic chamber as described above.

**Table 3:** **Uninoculated medium controls containing various concentrations of fatty acids and sulfide amended to represent crotonate metabolism by *S. aciditrophicus* strain SB or lactate metabolism by *Desulfovibrio sp*. strain G11**.

**Crotonate metabolism**	**Lactate metabolism**
20 mM crotonate	20 mM lactate
10 mM crotonate + 20 mM acetate + 10 mM sulfide	10 mM lactate + 10 mM acetate + 10 mM sulfide
0 mM crotonate + 40 mM acetate + 20 mM sulfide	0 mM lactate + 20 mM acetate + 20 mM sulfide
No amendment medium control	No amendment medium control

### Profilometry

At the end of the incubation, the coupons were recovered and localized corrosion damage was assessed with light profilometry (PS50 profilometer, Nanovea, Irvine, CA). The coupons were acid-cleaned to remove corrosion deposits according to ASTM Standard A576-90b (2006). The initial surface profiling of coupons was conducted by a cooperating external laboratory (Phillips 66, Bartlesville, OK, USA). However, a model PS50 profilometer (Nanovea, Irvine, CA) was eventually acquired for this purpose. Only the flat surface of the cylindrical coupons were analyzed for localized pitting evaluations. Line scans were run at a data acquisition rate of 2000 Hz using a 300 μm optical pen at a 3.0 μm step size. All surface analysis was performed using 3D analysis software (Ultra v6.2 Nanovea, Irvine, CA). Pitting was defined as damage that was 20 μm below the mean surface plane and at least a 20 μm in diameter. These experimental parameters were chosen based on the distribution of collected points across all coupons analyzed. Surface damage was also assessed by comparing histograms for each coupon. Each histogram consists of 2501 points collected from the profilometer. The *y* value indicates the depth (μm) of the points while the *x* value is the percentage of points at that particular depth.

### Statistical methods

All statistical analyses were done using R (The R Foundation for statistical computing). Treatment means were estimated by a Bayesian multilevel model that accounted for the dependencies among the 2501 points from each coupon. Using the MCMCglmm package (Hadfield, 2010) and vauge priors, eight parallel MCMC chains were run for 10,000 iterations after burn-in; the largest ***R*** was less than 1.001. Our primary model produced point and error estimates consistent with a frequentist multilevel model (using the lme4 package; Bates, 2010) and with a frequentist ANOVA (that considered only the mean of each coupon). Coupons not scanned at the University of Oklahoma were excluded from the analysis since a different profilometry protocol was employed and the resulting histograms were not comparable. In addition, a medium control coupon was similarly excluded since it had a damaged area on its surface (but no pits matching the experimental parameters were evident). To be entirely transparent, a comparison of the datasets with and without the outliers is depicted in the supplemental information (see Figure S21). To facilitate reproducibility, the code and profilometry data are available at https://github.com/LiveOak/LylesCarbonSteelCorrosion.

### Analytical methods

Crotonate loss was measured by HPLC (Dionex model IC-3000, Sunnyvale, CA) using an Alltech Prevail™ organic acid column (250 × 4.6 mm, particle size 5 μM; Grace, Deerfield, IL) and UV absorbance dector (Dionex model AD25, Sunnyvale, CA). The gradient pump was operated at a flow rate of 1.0 ml/min and mixed a mobile phase consisting of 60% (vol/vol) KH_2_PO_4_ (25 mM, pH 2.5) and 40% acetonitrile. The UV absorbance detector was set to 254 nm. Sulfate depletion was analyzed by ion chromatography (Dionex model IC-1000, Sunnyvale, CA) and has been previously described in Lyles et al. (2013). Methane production was monitored by gas chromatography (Packard model 427, Downers Grove, Ill.) and has been previously described in Gieg et al. (2008).

## Results

### Anaerobic hydrocarbon biodegradation and the corrosion of carbon steel

There are many important ecological consequences associated with anaerobic hydrocarbon biodegradation in the environment. We used pure cultures and defined co-cultures to investigate the impact of this metabolism on one of the most important ecological consequences, the biocorrosion of carbon steel. *D. alkanexedens* strain ALDC and *M. hungatei* strain JF-1 were cultured axenically and as a syntrophic partner in the presence of carbon steel coupons. Coupon damage was assessed by periodically measuring the instantaneous corrosion rate during the course of the experiment and by surface profilometry at the end of the incubation period (Table 4).

**Table 4:**
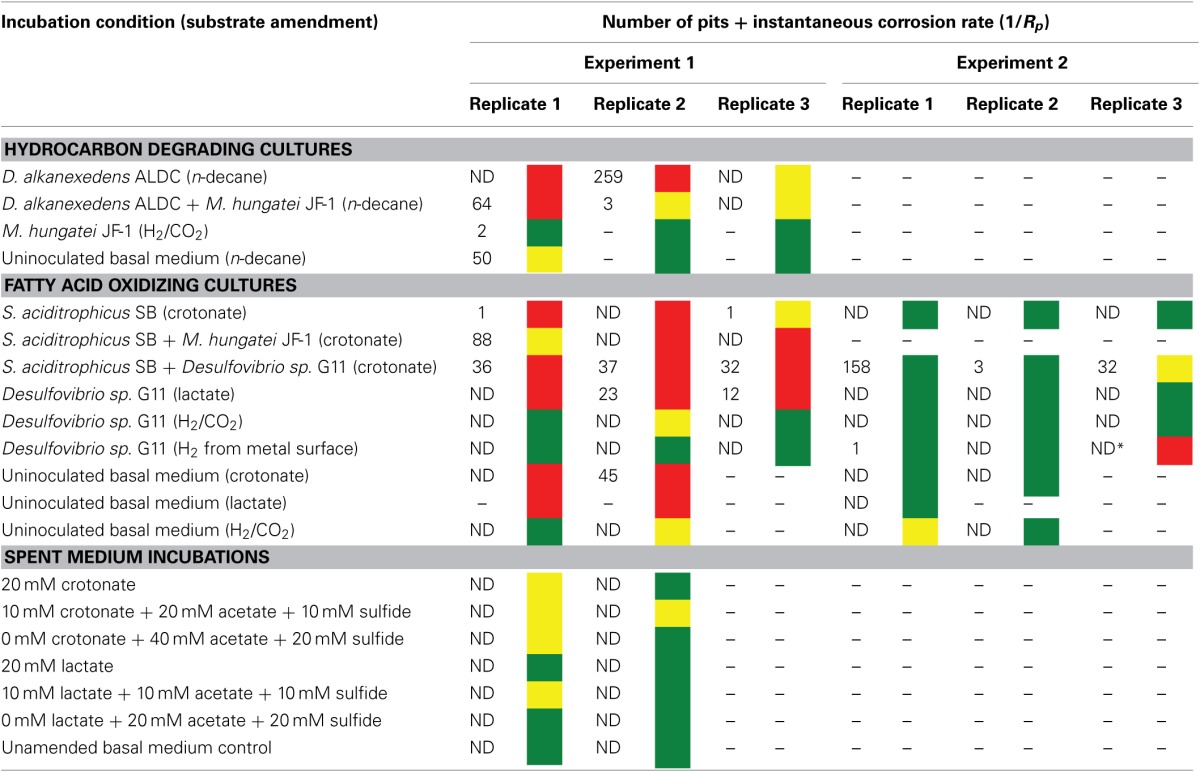
**The number of pits and the instantaneous corrosion rates for replicates of the hydrocarbon and fatty acid oxidizing cultures as well as the spent medium incubations**.

*ND, Not detectable*.

*“–”, Replicate not done or data not available*.

^*^The metal had pitting on the side but could not be quantified by the profilometer.

*1/R_p_ =* 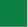 *10^−5^ ohms^−1^ cm^−2^, <0.0254 mmpy;* 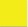 *10^−4^ ohms^−1^ cm^−2^, 0.0254–0.127 mmpy;* 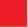 *10^−3^ ohms^−1^ cm^−2^, 0.127–0.508 mmpy.*

**Figure 2:**
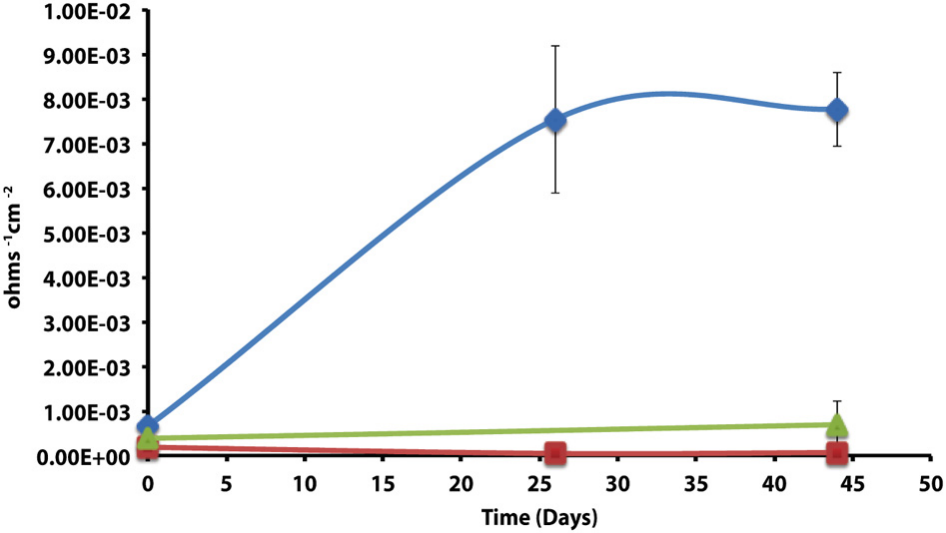
**Instantaneous corrosion rates (1/*Rp*) for pure cultures of *D. alkanexedens* strain ALDC (**
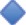
**) and *M. hungatei* strain JF-1 (**
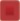
**) as well as the syntrophic co-culture (**
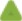
**) of the two microorganisms (standard deviation incubations *n* = 3, axenic *D. alkanexedens* strain ALDC incubations *n* = 2)**.

After 44 days of incubation, *D. alkanexedens* strain ALDC cultured on *n*-decane reduced sulfate at a rate of 0.060 ± 0.014 mM SO_4_•day^−1^ (Figure S5) and produced an instantaneous corrosion rate of 7.8 × 10^−3^ ± 8.3 × 10^−4^ ohms^−1^ cm^−2^ (~0.381 mmpy; Figure 2). Approximately 18% of the *n*-decane was oxidized during the incubation period based on the amount of sulfate reduced within the incubation (data not shown). When *D. alkanexedens* strain ALDC and *M. hungatei* strain JF-1 were co-cultured on the same hydrocarbon, an average of 62.0 ± 22.5 μmole methane (Figure S6) was produced, suggesting that ~0.2% of the parent hydrocarbon was utilized by the co-culture. The instantaneous corrosion rate for the co-culture was 7.0 × 10^−4^ ± 4.3 × 10^−4^ ohms^−1^ cm^−2^ (~0.0508 mmpy; Figure 2). Pure cultures of *M. hungatei* strain JF-1 cultured hydrogenotrophically (H_2_/CO_2_) produced ~1500 ± 700 μmole methane over the initial 24 days incubation but the rate decreased with each headspace exchange over the entire incubation (Figure S7). The pure methanogen exhibited an instantaneous corrosion rate of 6.56 × 10^−5^ ± 4.4 × 10^−5^ ohms^−1^ cm^−2^ (<0.0254 mmpy); a value that was even lower than the comparable rate determination in the uninoculated control (1.01 × 10^−4^ ± 4.48 × 10^−5^ ohms^−1^ cm^−2^; <0.0254 mmpy).

When profilometry was used to assess localized corrosion in the coupons, differences between replicate incubations were evident (Table 4; Figure S8). One coupon replicate in the *D. alkanexedens* strain ALDC incubation exhibited substantial pitting (259 pits) while pits were not evident on the other replicate coupon despite similar sulfate reduction (Figure S5) and instantaneous corrosion rates (Figure 2). Pitting was not as severe for coupon replicates incubated in the syntrophic co-culture. That is, 66 pits were counted on replicate one, 3 pits on replicate two, and 0 pits on replicate three. The pure culture incubation of *M. hungatei* strain JF-1 and the uninoculated control had 2 and 50 pits, respectively.

### Anaerobic fatty acid biodegradation and the corrosion of carbon steel

**Figure 3:**
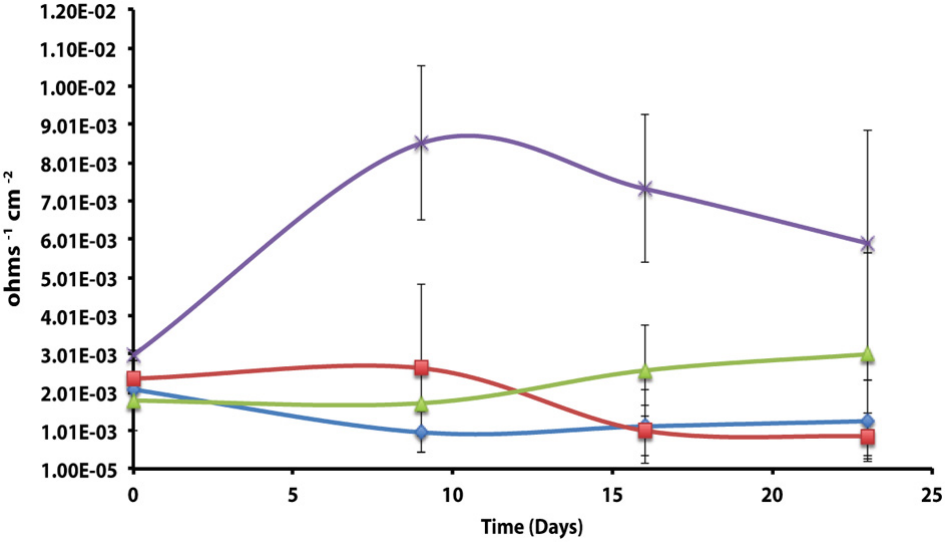
**Instantaneous corrosion rates (1/*Rp*) for pure cultures of *S. aciditrophicus* strain SB (**
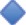
**) and syntrophic co-cultures with *M. hungatei* strain JF-1 (**
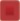
**) and *Desulfovibrio sp*. strain G11 (**
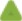
**) as well as an uninoculated basal medium controls amended with 20 mM of crotonate (**
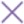
**) (standard deviation incubations *n* = 3, medium controls *n* = 2)**.

The corrosion potential of the fatty acid-oxidizing syntroph, *S. aciditrophicus* strain SB, was evaluated in a similar manner by growing the bacterium, as a pure culture or in defined co-culture with *M. hungatei* strain JF-1 or *Desulfovibrio sp*. strain G11 (Table 4; Figure 3). The instantaneous corrosion rates were not substantially different between the pure culture of *S. aciditrophicus* strain SB and co-cultures *M. hungatei* strain JF-1 or *Desulfovibrio sp*. strain G11. However, localized corrosion increased when *S. aciditrophicus* strain SB was co-cultured with *Desulfovibrio sp*. strain G11 compared to when the syntroph was grown as a pure culture or when it was coupled with the methanogen (Table 4). Additionally, localized corrosion was also evident when coupons were exposed to pure cultures of *Desulfovibrio sp*. strain G11 grown in lactate-amended incubations with sulfate as the electron acceptor.

The instantaneous corrosion rates for pure cultures of *S. aciditrophicus* strain SB and related co-cultures are compared in Figure 3. Incubations containing axenic cultures of *S. aciditrophicus* strain SB were found to produce an instantaneous corrosion rate of 1.12 × 10^−3^ ± 9.65 × 10^−4^ ohms^−1^ cm^−2^ (~0.0762 mmpy). In co-culture with *M. hungatei* strain JF-1, 15.65 ± 7.16 μmole methane was produced over the 16 days incubation period (Figure S9), and the instantaneous corrosion rate was 2.64 × 10^−3^ ± 2.91 × 10^−3^ ohms^−1^ cm^−2^ (~0.178 mmpy). When co-cultured with *Desulfovibrio sp*. strain G11, the sulfate reduction rate was 0.34 ± 0.08 mM SO_4_•day^−1^ (Figure S10), and the instantaneous corrosion rate was 2.6 × 10^−3^ ± 1.19 × 10^−3^ ohms^−1^ cm^−2^ (~0.1524 mmpy). Uninoculated basal medium controls amended with 20 mM of crotonate had an instantaneous corrosion rate of 7.33 × 10^−3^ ± 2.01 × 10^−3^ ohms^−1^ cm^−2^ (~0.4318 mmpy). In all incubations containing *S. aciditrophicus* strain SB, crotonate was not detected by HPLC after 14 days of incubation (Figure S11). Additionally, the large standard deviations observed between 1/*R_p_* values (Figure 3) associated with incubations of *S. aciditrophicus* strain SB and related co-cultures suggest that there is no difference in instantaneous corrosion rates, despite the formation of different metabolic end products (i.e., sulfide, methane, acetate) within each of the various incubation conditions.

**Figure 4:**
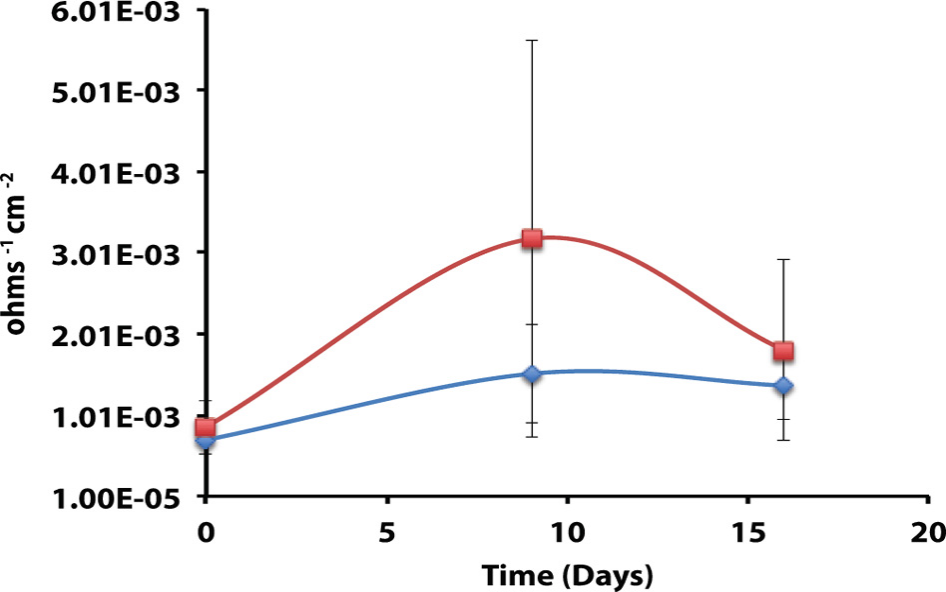
**Instantaneous corrosion rates (1/*Rp*) for lactate-amended pure cultures of *Desulfovibrio sp*. strain G11 (**
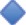
**) and uninoculated basal medium controls amended with 20 mM lactate (**
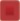
**) (standard deviation incubations *n* = 3, medium controls *n* = 2)**.

**Figure 5:**
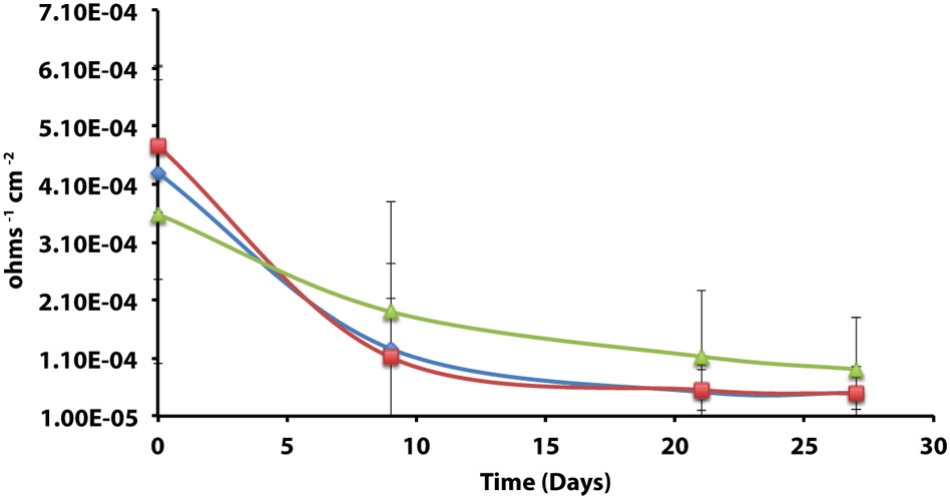
**Instantaneous corrosion rates (1/*Rp*) for axenic incubations of *Desulfovibrio sp*. strain G11 cultured autotrophically on 138 kPa of H_2_/CO_2_ (**
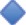
**) and hydrogen from the metal surface (**
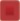
**), as well as an uninoculated basal medium controls (**
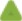
**) amended with 138 kPa of H_2_/CO_2_ or N_2_/CO_2_ (standard deviation incubations *n* = 3, medium controls *n* = 2)**.

Axenic incubations of *Desulfovibrio sp*. strain G11 cultured in the same media but amended with 20 mM of lactate produced an instantaneous corrosion rate of 1.5 × 10^−3^ ± 6.0 × 10^−4^ ohms^−1^ cm^−2^ (~0.089 mmpy; Figure 4). These incubations reduced sulfate at a rate of 2.00 ± 0.1 mM SO_4_•day^−1^ (Figure S10). The uninoculated media control containing 20 mM of lactate produced an instantaneous corrosion rate of 3.2 × 10^−3^ ± 6.0 × 10^−4^ ohms^−1^ cm^−2^ (~0.1778 mmpy; Figure 4). Thus, there is no significant difference for the instantaneous corrosion rates between *Desulfovibrio sp*. strain G11 and uninoculated control incubations. Additionally, when *Desulfovibrio sp*. strain G11 was cultured autotrophically with 138 kPa overpressure of H_2_/CO_2_ in the headspace as well as from hydrogen emanating from the metal surface, the sulfate reduction rate was 0.79 ± 0.04 mM SO_4_•day^−1^ and 0.35 ± 0.10 mM SO_4_•day^−1^, respectively (Figure S10). In all replicates of autotrophically-grown cultures of this microorganism, the instantaneous corrosion rates (Figure 5) ranged between 5.17 × 10^−5^ ± 4.70 × 10^−5^ and 5.51 × 10^−5^ ± 3.51 × 10^−5^ ohms^−1^ cm^−2^ (<0.0208 mmpy), which is lower than the uninoculated controls containing either 138 kPa of H_2_/CO_2_ or N_2_/CO_2_ in the headspace (1.02 × 10^−4^ ± 1.13 × 10^−4^ ohms^−1^ cm^−2^).

Carbon steel coupons were assessed for localized corrosion using profilometry, and the numbers of pits counted from surface profiles (Figure S12) are compared in Table 4 (experiment 1). Even though pure cultures of *S. aciditrophicus* strain SB produced instantaneous corrosion rates of 10^−3^ and 10^−4^, only 2 pits were identified on the metal surface. When *S. aciditrophicus* strain SB was co-cultured with *Desulfovibrio sp*. strain G11, an average of 35 ± 2.6 pits were identified across the three replicates. Finally, when *S. aciditrophicus* strain SB was co-cultured with *M. hungatei* strain JF-1, one replicate produced 88 pits, while the other replicates had no detectable pits. When coupons were exposed to pure cultures of *Desulfovibrio sp*. strain G11 grown on lactate, replicate one had no pits, but replicates two and three had 23 and 12 pits, respectively. No pits were detected on coupons analyzed from incubations containing *Desulfovibrio sp*. strain G11 cultured under autotrophic conditions. Additionally, exposure to uninoculated medium amended with 20 mM crotonate caused one out of two metal samples to produce 45 pits.

Given the unexpectedly high level of variability encountered, the same experiments were repeated with the same degree of replication. With few exceptions, the instantaneous corrosion rates were largely in the 10^−5^ ohms^−1^ cm^−2^ range (Table 4 experiment 2; Figure S13), and pitting was mainly associated with the *S. aciditrophicus* strain SB and *Desulfovibrio sp*. strain G11 co-cultures (Table 4 experiment 2; Figure S14). *S. aciditrophicus* strain SB alone or in co-culture with *Desulfovibrio sp*. strain G11 metabolized ~2 mM crotonate over the 30 days incubation (Figure S15), and the co-culture incubation reduced sulfate at 0.30 ± 0.08 mM SO_4_•day^−1^ (Figure S16). For comparison, *Desulfovibrio sp*. strain G11 cultured with lactate, H_2_/CO_2_ overpressure, or hydrogen emanating from the coupon reduced sulfate at 1.08 ± 0.006, 0.36 ± 0.06, and 0.16 ± 0.09 mM SO_4_•day^−1^, respectively (Figure S15). Thus, even though similar sulfate reduction rates were measured between cultures and co-cultures from experiment 1, the instantaneous corrosion rates as well as localized corrosion were substantially less within these repeat incubations (Table 4).

### Corrosivity of spent media

The apparent corrosivity of uninoculated controls (Table 4) caused us to question the role of parent substrates and metabolic end products in exacerbating coupon damage. This prompted an experiment wherein acetate and sulfide were exogenously added to the sterile basal medium to simulate the concentration of these substances following the metabolism of crotonate by a co-culture of *S. aciditrophicus* strain SB and *Desulfovibrio sp*. strain G11. The same thing was done to simulate the products acetate and sulfide associated with lactate metabolism by *Desulfovibrio sp*. strain G11. These incubation conditions were chosen because localized corrosion was consistently associated with the production of acetate and sulfide. The results indicated that instantaneous corrosion rates typically ranged between 10^−4^ and 10^−5^ ohms^−1^ cm^−2^, and pitting was not detected on any of the metal surfaces (Table 4; Figures S17–S20).

### Statistical tests

**Figure 6:**
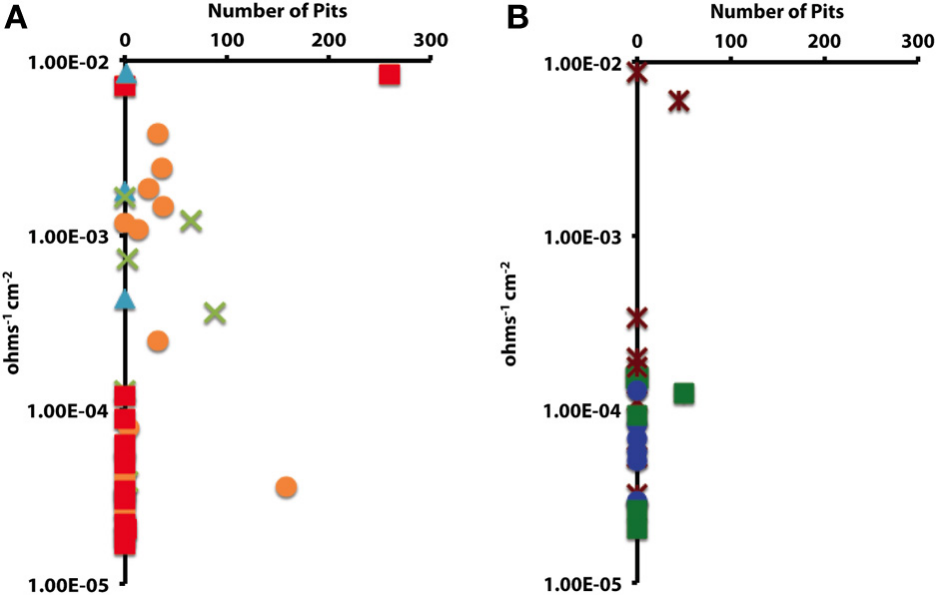
**The impact of (A) microbial cultures producing sulfide and acetate (**
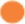
**), sulfide only (**
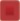
**), acetate only (**
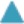
**), and methane (**
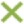
**) on localized corrosion**. Compared to **(B)** uninoculated medium controls amended with crotonate (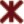), lactate (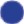), or no VFA (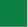).

**Figure 7:**
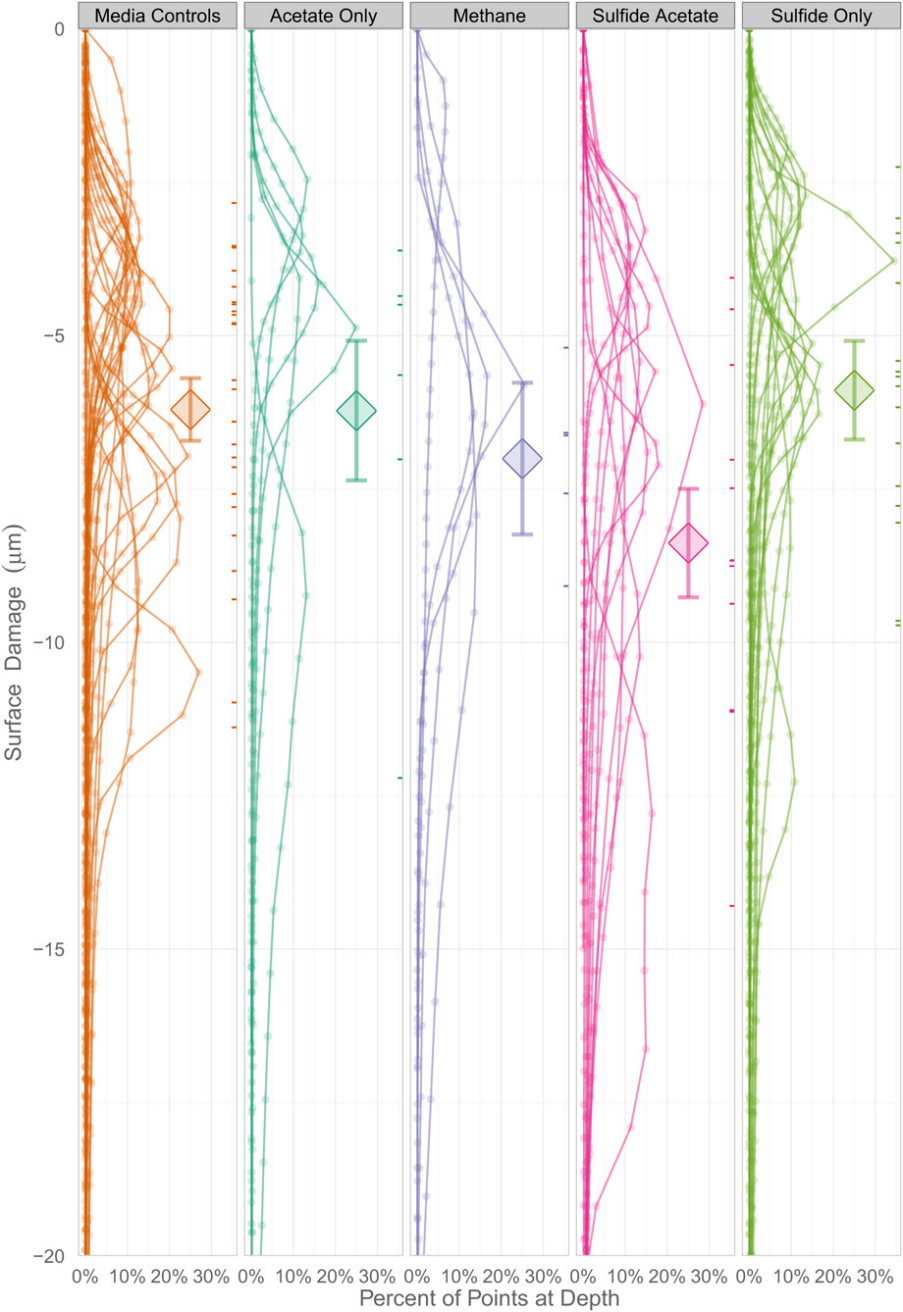
**A Bayesian multilevel model comparing the mean surface damage for treatment groups as well as individual coupons**. The diamonds indicate a treatment group's mean depth, and the tick marks on the right side of the columns indicate an individual coupon's mean depth. The error bars mark the 16 and 84% percentiles of each treatment's posterior distribution, which corresponds to the asymptotic 68% coverage of a ±1 standard error band, but is allowed to be asymmetric.

In order to determine whether statistical differences existed between the various experimental treatments, incubations were grouped according to end product production. These treatment groups included: sulfide only, sulfide and acetate, acetate only, methane, and uninoculated media controls. The treatment groups were then assessed by plotting the number of pits vs. the instantaneous corrosion rates for the individual microbial incubations (Figure 6A), and then compared to the uninoculated media controls (Figure 6B). The results suggested that when sulfide and acetate were produced as end products, corrosion was elevated relative to other microbial incubations as well as to the media controls (Figure 6). To further examine these differences, the surface damage for each coupon was compared using a Bayesian multilevel model. Figure 7 displays the 63 histograms of points collected from the profilometer (i.e., one histogram for each coupon) separated into the five treatment groups. A histogram that is concentrated near the top of a panel (e.g., −5 μm) indicates less surface damage than a histogram concentrated closer to the bottom (e.g., −20 μm). A value of 0 μm represents the highest point on the coupon's surface. The histograms are skewed down, indicating that the depth of the (numerous) points are greater than the height of the (infrequent) peaks. The results indicated that incubations producing both sulfide and acetate as end products was the only treatment group that corroded significantly more than the media controls (*p* = 0.016); typically the mean surface damage for these coupons was 2.2 μm deeper than the uninoculated media controls.

## Disscussion

Anaerobic hydrocarbon degradation is an important ecological process within petroleum-laden environments, and one potential consequence of this metabolic activity is the sulfide-induced corrosion of carbon steel. However, the inherent complexity of the natural environment often makes it difficult to ascertain the biotic and abiotic factors that substantially influence biocorrosion. Our approach was to investigate this process in a more defined manner. That is, biocorrosion can be viewed as the interaction between electron donors of interest, a specific inoculum, the prevailing environmental conditions, and the composition of the metal (Suflita et al., 2013). Thus, we used defined microbial systems wherein the biological interactions with the electron donors were known and the metabolic end products could be reasonably anticipated. More specifically, we explored the impact of sulfate as an electron acceptor on metal biocorrosion in defined microbial assemblages and compared the damage to coupons when the same organisms were cultivated as syntrophic partnerships.

The corrosivity of the alkane-degrading, sulfate-reducing bacterium *D. alkanexedens* strain ALDC was previously assessed and was found to produce higher instantaneous corrosion rates and more pits compared to an uninoculated medium control containing 20 mM sulfide (Suflita et al., 2013). Presumably, the production of sulfide by *D. alkanexedens* strain ALDC from *n*-decane oxidation was responsible for the increase in instantaneous corrosion rate and localized corrosion. In this study, when axenic incubations of *D. alkanexedens* strain ALDC were cultured on *n*-decane under sulfate-reducing conditions, instantaneous corrosion rates were ~10 times higher than comparable pure cultures of *M. hungatei* strain JF-1 grown on H_2_/CO_2_ or in a co-culture of the two microorganisms (Figure 2). Pitting also decreased when coupons were incubated with *M. hungatei* strain JF-1 alone or with the co-culture (Table 4; Figure S8). The biological replicates of *D. alkanexedens* strain ALDC pure culture incubations had similar sulfate reduction and instantaneous corrosion rates (Figure 2; Figure S5); however, despite these similarities, the replicate incubations had a high variability in the number of pits observed on the individual coupons (Table 4). This result suggests that the coupons were differentially pitting even though the replicate incubations were biologically and chemically similar. Variability in localized corrosion was also observed in both the co-culture and the uninoculated control incubations, in which a single replicate produced pits under the respective incubation conditions (Table 4). This result suggested that pitting was not specifically a function of sulfide production, as this end product was not produced in either incubation. Nevertheless, coupon pitting under methanogenic conditions as well as with uninoculated controls was a relatively rare occurrence compared to incubations under sulfate-reducing conditions (Figures 6, 7; Figure S21).

Previous research has suggested that the acetate concentration can exacerbate carbon steel corrosion by multiple mechanisms (Crolet et al., 1999; Hedges and McVeigh, 1999; Garsany et al., 2003; Suflita et al., 2008). At less than circumneutral pH values, at least some fraction of the acetate will exist as acetic acid and cause direct damage to metal surfaces. Therefore, it may be that *S. aciditrophicus* strain SB can contribute to corrosion through the production of acetate from the metabolism of fatty acids as a pure culture or when in syntrophic partnership. However, the instantaneous corrosion rates were not significantly different between axenic cultures of *S. aciditrophicus* strain SB and co-culture incubations with *M. hungatei* strain JF-1 or *Desulfovibrio sp*. strain G11 (Figure 3). However, when the coupons were subject to profilometric analysis, pitting was largely restricted to co-culture incubations of *S. aciditrophicus* strain SB and *Desulfovibrio sp*. strain G11 (Table 4 experiment 1; Figure S12). When *S. aciditrophicus* strain SB and *Desulfovibrio sp*. strain G11 co-culture incubations were repeated, instantaneous corrosion rates were substantially slower (Table 4 experiment 2; Figure S13), but pits were identified on all metal samples from the incubations (Table 4 experiment 2; Figure S14). Despite the 18 mM difference in crotonate metabolism between the experiments (Figures S11, S15), the *S. aciditrophicus* strain SB and *Desulfovibrio sp*. strain G11 co-culture was the only incubation condition that produced localized corrosion in all six replicates. The results suggest that corrosion is likely increased due to the production of both acetate and sulfide during the metabolism of crotonate under sulfate-reducing conditions.

The impact of lactate-grown *Desulfovibrio sp*. strain G11 on metal corrosion was similarly evaluated. This organism incompletely oxidizes lactate to acetate and uses sulfate as an external electron acceptor. Thus, if localized corrosion is at least a function of both acetate and sulfide production, pitting would be expected on the metal coupons. In fact, the results are largely consistent with this contention in that pitting occurred on metal surfaces in two of three replicate incubations (Table 4 experiment 1; Figure S12). Nevertheless, when these incubations were repeated, the instantaneous corrosion rates were substantially lower and no pits were evident by profilometry (Table 4 experiment 2; Figure S14) even though similar rates of sulfate reduction were measured between the two experiments (Figures S10, S16). The variability in pitting behavior between replicate incubations and repeat experiments that exhibited similar rates of substrate utilization and metabolic end product formation is enigmatic. Since the biological activity and the resulting chemistry is both defined and controlled, we are forced to attribute the variability to incubation components we could not adjust. More specifically, we presume the variations can somehow be attributed to differences in the composition of the carbon steel coupons. However, according to the bulk analysis by the manufacturer, the coupons were compositionally similar (Table S1).

To exemplify, autotrophically cultured *Desulfovibrio sp*. strain G11 cultures are able to reduce sulfate at different rates depending on whether the coupon itself served as a hydrogen source or if the electron donor was supplied exogenously. In the latter case, the sulfate reduction rate was comparable to the co-culture of this organism with *S. aciditrophicus* strain SB (Figures S10, S16). However, corrosion measures were substantially less with the pure culture relative to the co-culture, thus implicating acetate as a confounding factor in sulfide-induced corrosion. (Table 4 experiments 1 and 2). The only exception is one replicate from experiment two (Table 4), which produced an instantaneous corrosion rate of 10^−3^ ohms^−1^ cm^−2^ and had pitting on the side of the coupon (data not shown). Thus, only one metal coupon out of twelve substantially corroded upon exposure to autotrophically-grown *Desulfovibrio sp*. strain G11. Considering the relatively small amount of sulfide produced in these incubations (~2 mM), the lack of acetate production, and the relatively consistent levels of coupon corrosion, differences in the corrosion behavior of a single replicate also point to potential differences in the coupons themselves.

Methanogens such as *Methanobacterium thermoautotrophicum* (Lorowitz et al., 1992) and *Methanococcus maripaludis* strain KA1 (Uchiyama et al., 2010) have been previously described to stimulate corrosion by either scavenging hydrogen or direct electron utilization from the surface of the metal coupons, respectively. *M. hungatei* strain JF-1 has not been reported to utilize either of these corrosion mechanisms, and when the metal was the only source of hydrogen in the incubation, methane production, instantaneous corrosion rates, and pitting were all negligible (data not shown) over the incubation period. Additionally, incubations of *M. hungatei* strain JF-1 amended with 138 kPa of H_2_/CO_2_ did not exacerbate corrosion on any metal sample (Table 4). However, *M. hungatei* strain JF-1 was associated with corrosion within two incubations. The first was in co-culture with *D. alkanexedens* strain ALDC (Table 4, replicate 1, 64 pits; Figure S8), and the second was in co-culture with *S. aciditrophicus* strain SB (Table 4 experiment 1, replicate 1, 88 pits; Figure S12). Considering methane production was similar between replicate incubations (Figures S6, S9), the lack of sulfide production, and that pits were not detected on any other metal samples, the corrosion of these particular coupons are also attributed to the variability in the elemental composition of the metal sample.

Uninoculated media controls containing crotonate and lactate occasionally produced higher instantaneous corrosion rates (Figures 2, 3) than the corresponding inoculated incubations, and one replicate of a crotonate exposed coupon was found to have 45 pits at the end of the incubation (Table 4 experiment 1; Figure S12). Thus, we concluded that the potential for metal corrosion due to exposure to various fatty acids could be important considering that formate, acetate, propionate, butyrate, and benzoate have all been detected in oil reservoirs with concentrations exceeding 20 mM (Magot et al., 2000). However, when comparable uninoculated controls were specifically evaluated, corrosion was found to be negligible (Table 4 experiment 2). Additionally, corrosion was not stimulated on metal samples that were exposed to uninoculated incubations amended with crotonate, lactate, acetate and sulfide to represent the spent medium of *S. aciditrophicus* strain SB and *Desulfovibrio sp*. strain G11 co-cultures as well as lactate-amended *Desulfovibrio sp*. strain G11 pure cultures (Table 4; Figures S17–S20). Thus, the differences between our initial observations and subsequent experimentation (Table 4) also seem to be a function of the variable nature of the coupons, despite identical manufacturer bulk analyses.

Biocorrosion experiments exhibited high standard deviations for instantaneous corrosion rates and pitting for metal samples that was independent of inoculum type, biomass levels, initial substrate concentration, rates of microbial activity, or the degree of end product formation. Eliminating these factors as major contributors to the measured variability, forces us to attribute the large standard deviations to inconsistencies in the individual metal coupon samples. The bulk analysis of the metal from the manufacturer does not lend credence to this suggestion. However, low grade carbon steel is known to contain manganese sulfide inclusions (MnS) that are sites for pitting initiation and pit propagation (Vuillemin et al., 2003; Avci et al., 2013). The density of these submicron sized inclusions on carbon steel is typically thousands per square millimeter (Avci et al., 2013). It is unknown if the inclusions are evenly (randomly) distributed in metal or if they are clustered (heterogeneous) to any degree. It also seems clear that some inclusions are more susceptible as sites of pit initiation than others (Wranglen, 1974; Davis, 2013). Thus, both the distribution and the reactivity of inclusion bodies within a metal sample could substantially influence corrosion processes and help explain why seemingly similar metal samples may behave differently in corrosion experiments.

There was generally no agreement between instantaneous corrosion rates and the number of pits; that is localized corrosion did not always occur on coupons with higher (10^−3^ ohms^−1^ cm^−2^) 1/*R_p_* values (Table 4; Figure 6). Electrochemical measurements, such as LPR, are considered highly sensitive and accurate (<0.5% error; Jones, 1996) for monitoring generalized corrosion, and profilometry can detect and quantify pits on a micron-scale. However, considering these methods were not always in agreement, other corrosion analyses would seem pertinent (e.g., weight loss, total iron determinations, manganese loss). Additionally, by differentially focusing our analysis on bulk fluids, the findings may not reflect reactions occurring in a biofilm on the metal surface. Localized metabolic activities in microbial biofilms, particularly the production of organic acids, can cause the pH to substantially decrease at the metal surface (Vroom et al., 1999). These points notwithstanding, our results generally show that localized corrosion was elevated when coupons were exposed to sulfide-producing cultures relative to methanogenic cultures or to uninoculated controls (Figures 6, 7; Figure S21).

The major implications of this work are that the anaerobic biodegradation of hydrocarbons or associated fatty acid intermediates linked to sulfate reduction can have important consequences with respect to the biocorrosion of carbon steel. That is, when sulfate is available as an electron acceptor, microbial assemblages will produce sulfide and low molecular weight organic acids that generally increase the corrosion of carbon steel. However, when the same organisms are in sulfate-limited environments and forced to live a syntrophic existence with a methanogen, biocorrosion is substantially reduced. These trends must be considered fairly general with more specific inferences being somewhat masked by the surprisingly high degree of variability associated with the corrosion assessments. Since the biological and chemical characteristics of the incubations were controlled, we were forced to attribute the high variability to differences with the metal samples themselves.

## Author contributions

All authors contributed equally to this work. Christopher N. Lyles was responsible for designing electrochemical and profilometry analyses to assess the corrosion of metal coupons, as well as data analysis, interpretation of results, and drafting the manuscript. Huynh M. Le cultured the microorganisms and also contributed to data analysis and interpretation. William Howard Beasley provided the statistical analyses. Michael J. McInerney critically revised the manuscript as well as provided much needed insight into a variety of aspects surrounding syntrophic microorganisms. Joseph M. Suflita is the corresponding author and has overseen all aspects of this project for both scientific accuracy and integrity.

### Conflict of interest statement

The authors declare that the research was conducted in the absence of any commercial or financial relationships that could be construed as a potential conflict of interest.
