# Supplementary material for: Anaerobic hydrocarbon and fatty acid metabolism by syntrophic bacteria and their impact on carbon steel corrosion
Source: Front Microbiol. 2014 Apr 1;5:114. doi: 10.3389/fmicb.2014.00114 (PMC3978324; doi:10.3389/fmicb.2014.00114)
Supplement: Supplementary file 1 [file Presentation1.PDF]

Supporting Information

Anaerobic hydrocarbon and fatty acid metabolism by syntrophic bacteria and their impact on carbon steel corrosion

Christopher N. Lyles, Huynh M. Le, William H. Beasley, Michael J. McInerney, and Joseph M. Suflita

23 pages total, 1 table, and 21 figures

Table S1: Elemental composition of C1020 steel coupons used within this study. Two lots of the same type of metal samples were ordered and both had the exactly the same chemical and physical test report containing this elemental composition table. Lot one coupons were used in the *D. alkanexedens* strain ALDC incubations as well as the first experiment of *S. aciditrophicus* strain SB incubations. Lot two coupons were used in the repeat *S. aciditrophicus* strain SB experiment and also for the spent medium incubations.

C	Mn	P	S	Si	Cu	Ni	Cr	Mo	V	Nb	N	Sn	Al	Ti	Ca	Zn	Co
.20	.58	.008	.016	.20	.25	.12	.12	.017	.025	.002	.0091	.008	.003	.00100	.00140	.00100	.008

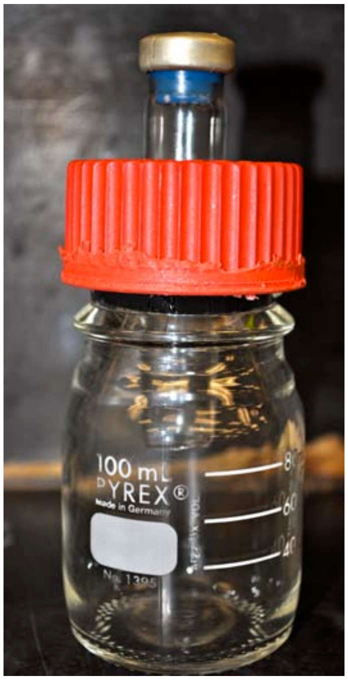

Figure S1: A 100 ml culture bottle used as an electrochemical cell during corrosion experiments with *D. alkanexedens* strain ALDC and *S. aciditrophicus* strain SB pure cultures and related co-cultures.

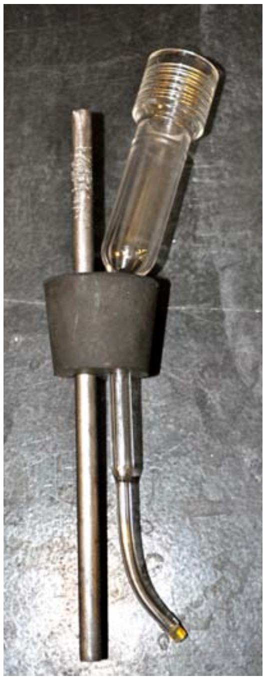

Figure S2: A number six-rubber stopper modified to hold a graphite counter electrode and a luggin probe (bridge tube) for the saturated calomel reference electrode.

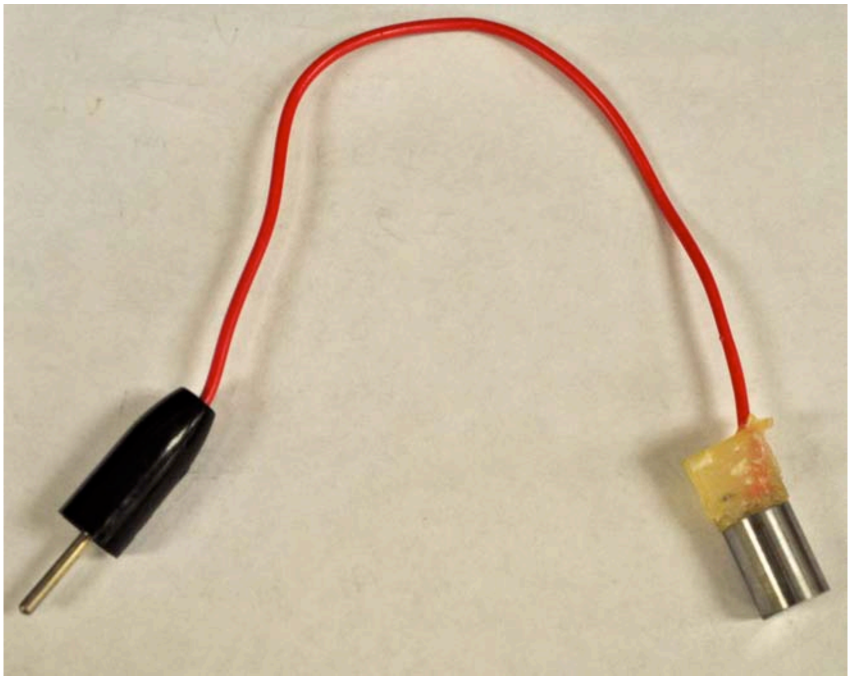

Figure S3: A C1020 working electrode. The wire was attached to the coupon using rosin core solder and epoxy sealed to prevent galvanic corrosion. The electrode pin (black) connects to the corresponding alligator clip from the potentiostat. This was incubated within the culture bottles during the experimental time.

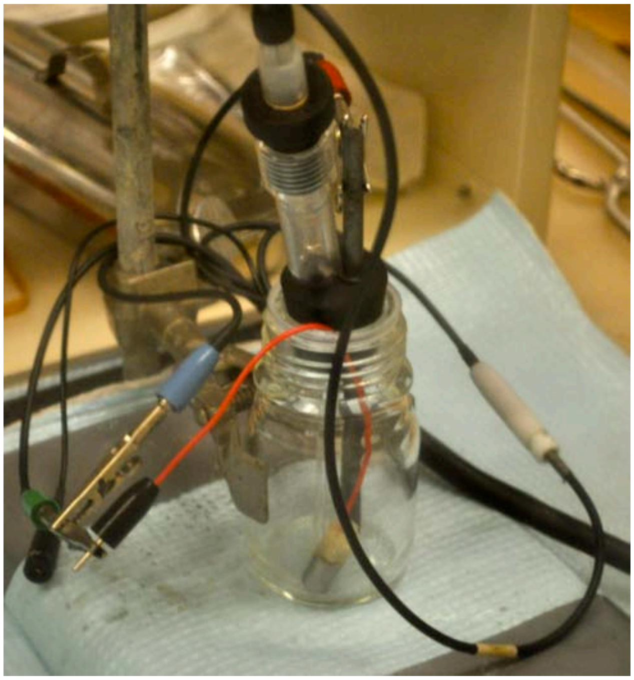

Figure S4: Complete electrochemical cell assembly inside the anaerobic chamber. Culture bottles were periodically brought into the anaerobic chamber and opened so that the sterile stopper assembly (Figure S2) could be placed inside the incubation. Corrosion was monitored using LPR curves taken every 5 minutes for a 30 minute period. Once complete, the culture bottles were resealed with sterile stoppers and the headspace exchanged to the appropriate atmosphere.

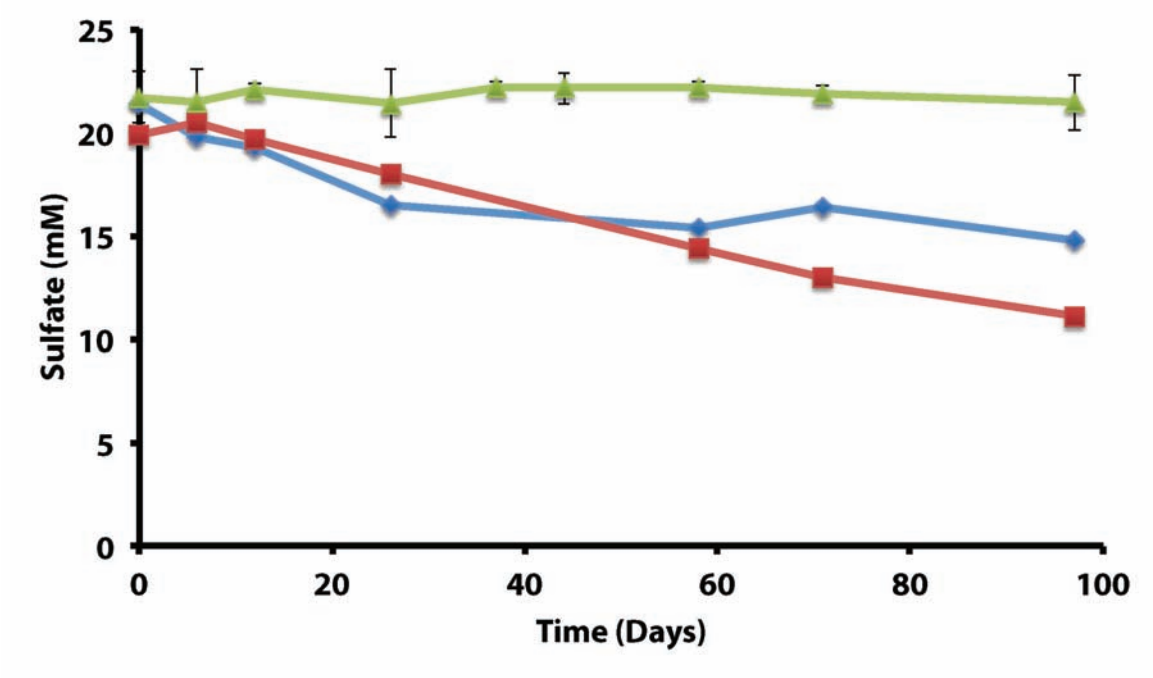

Figure S5: Sulfate reduction observed in axenic incubations of *D. alkanexedens* strain ALDC cultures replicates one (◆) and two (■). No sulfate loss was observed in uninoculated medium controls (▲; standard deviation *n*=3)

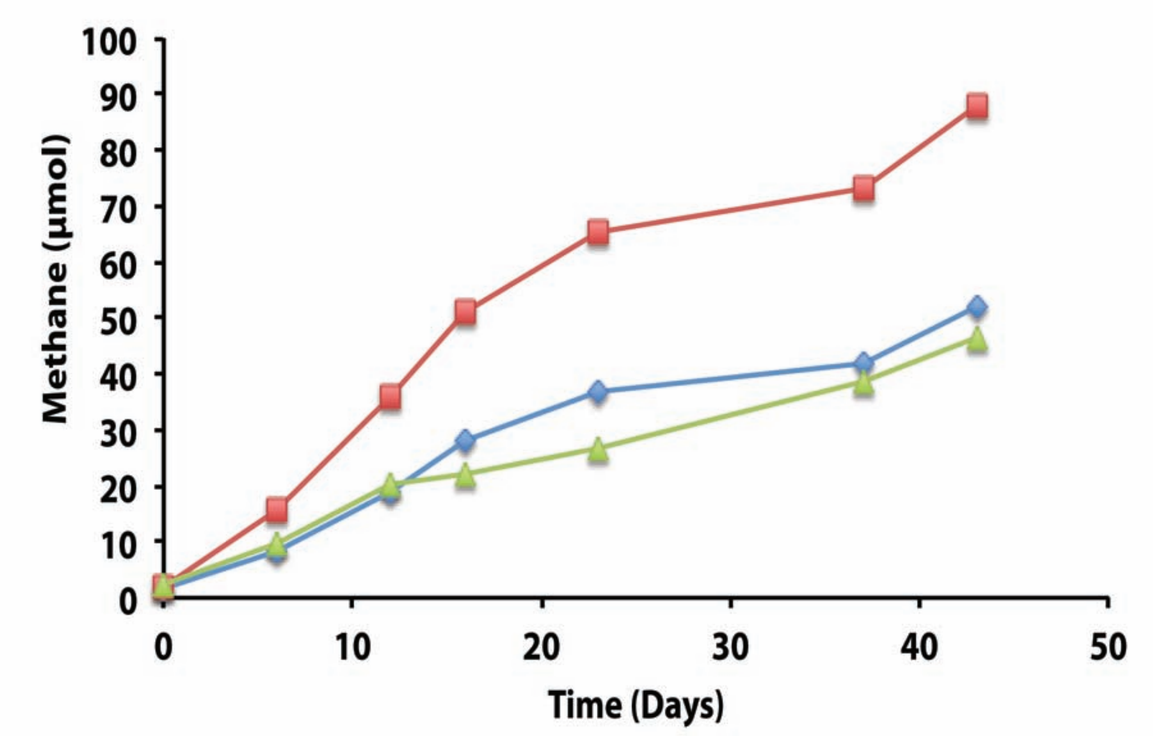

Figure S6: Methane production observed in co-culture incubations of *D. alkanexedens* strain ALDC and *M. hungatei* strain JF-1. Replicate 1 (◆) Replicate 2 (■) Replicate 3 (▲)

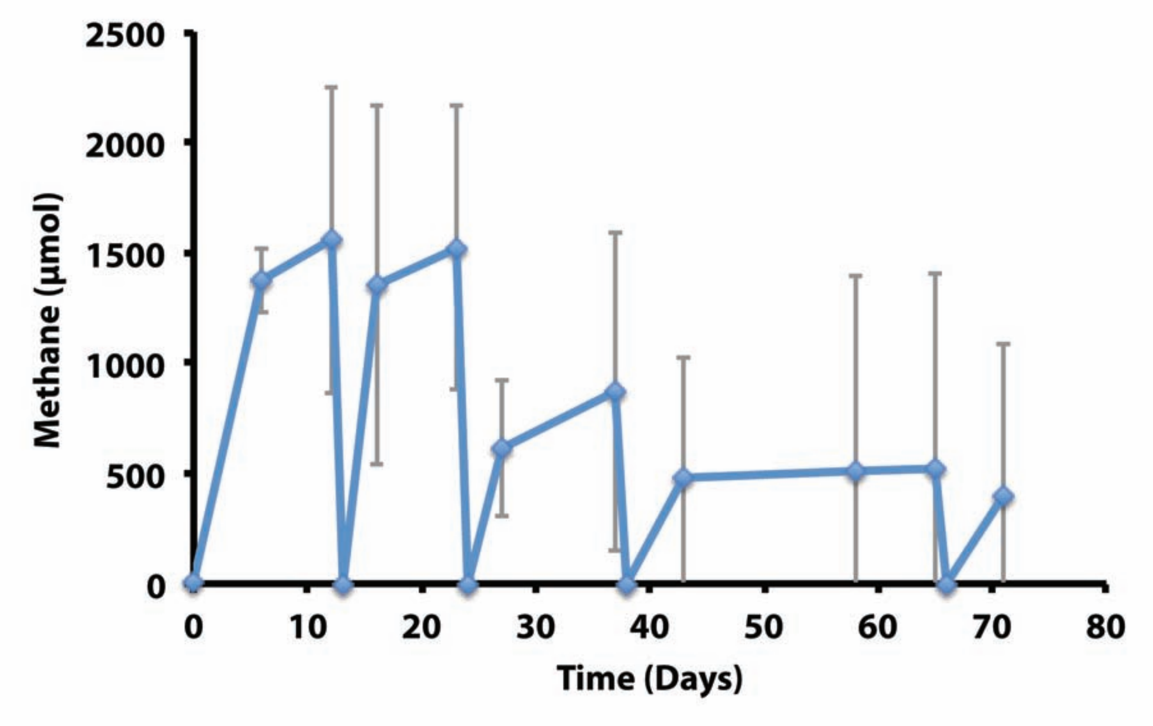

Figure S7: Methane production from pure cultures of *M. hungatei* strain JF-1 (◆) amended with 138 kPa of H_2/CO_2. The methane decreased to zero when the headspace was periodically exchanged and repressurized with 138 kPa of H_2/CO_2. Methane was not detected in uninoculated controls (standard deviation *n*=3).

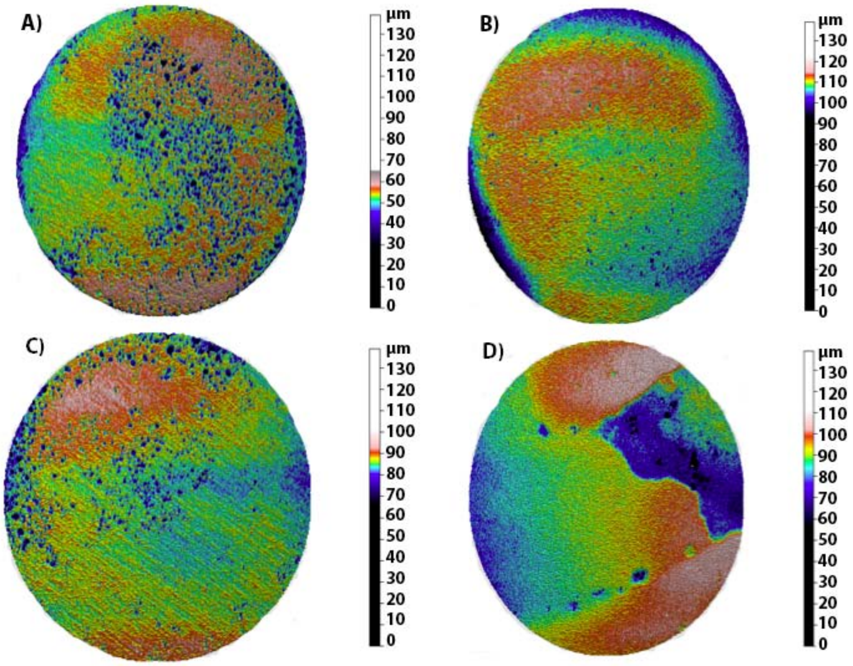

Figure S8: Surface profiles of C1020 metal coupons exposed to pure cultures of A) *D. alkanexedens* strain ALDC B) *M. hungatei* strain JF-1 as well as a C) syntrophic co-culture of the two microorganisms and a D) uninoculated media culture. The profilometry scanning was done at Phillips 66 (Bartlesville, OK, USA) and the surface analysis was done at the University of Oklahoma. The scale bar represents surface points ranging from the highest (white) to lowest (black) for each image.

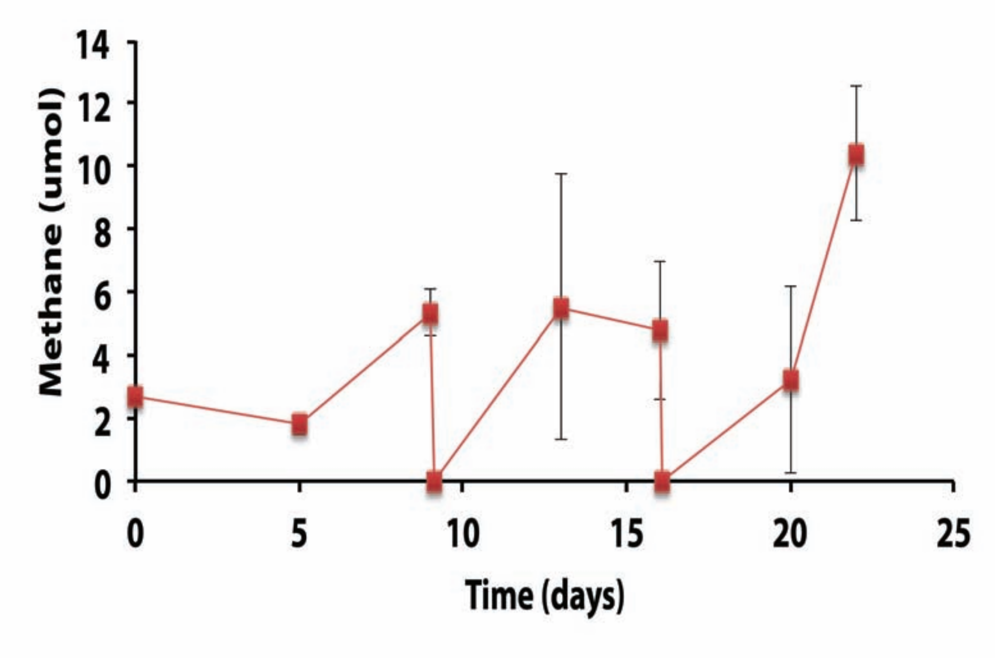

Figure S9: Methane production from co-cultures of *S. aciditrophicus* strain SB and *M. hungatei* strain JF-1 (■). Methane reached zero when cultures were opened to take LPR measurements within the anaerobic chamber. Methane was not detected in uninoculated controls (standard deviation *n*=3).

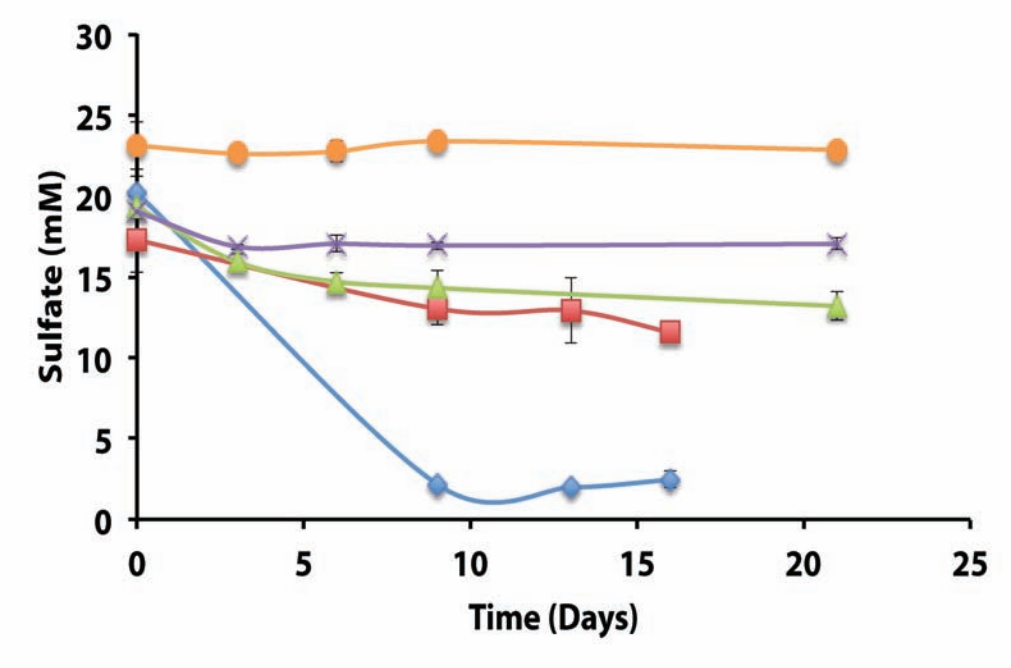

Figure S10: Sulfate reduction observed during experiment one in axenic *Desulfovibrio sp.* strain G11 cultures amended with lactate (◆), 138 kPa of H_2/CO_2 (▲), hydrogen from the metal surface (×), and co-cultured with *S. aciditrophicus* strain SB (■). Uninoculated basal medium controls (●) were also monitored but no sulfate depletion was observed (standard deviation n=3).

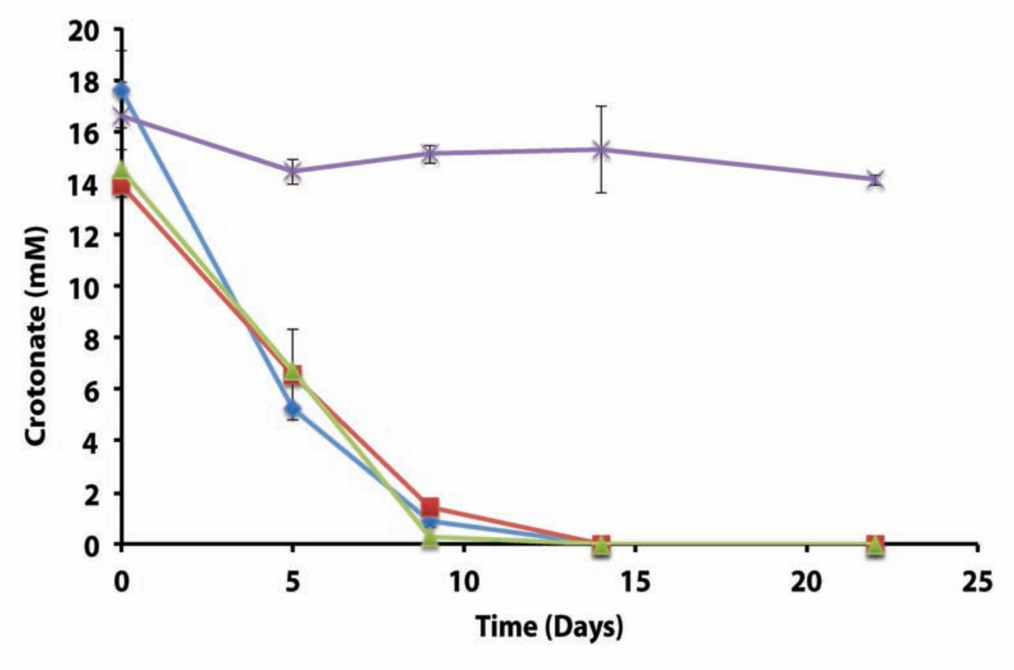

Figure S11: Crotonate depletion observed during experiment one in *S. aciditrophicus* strain SB incubations cultured axenically on crotonate (◆), in co-culture with *M. hungatei* strain JF-1 (■), and in co-culture with *Desulfovibrio sp.* strain G11 (▲). No crotonate loss was observed in uninoculated basal medium controls (×) (standard deviation *n*=3).

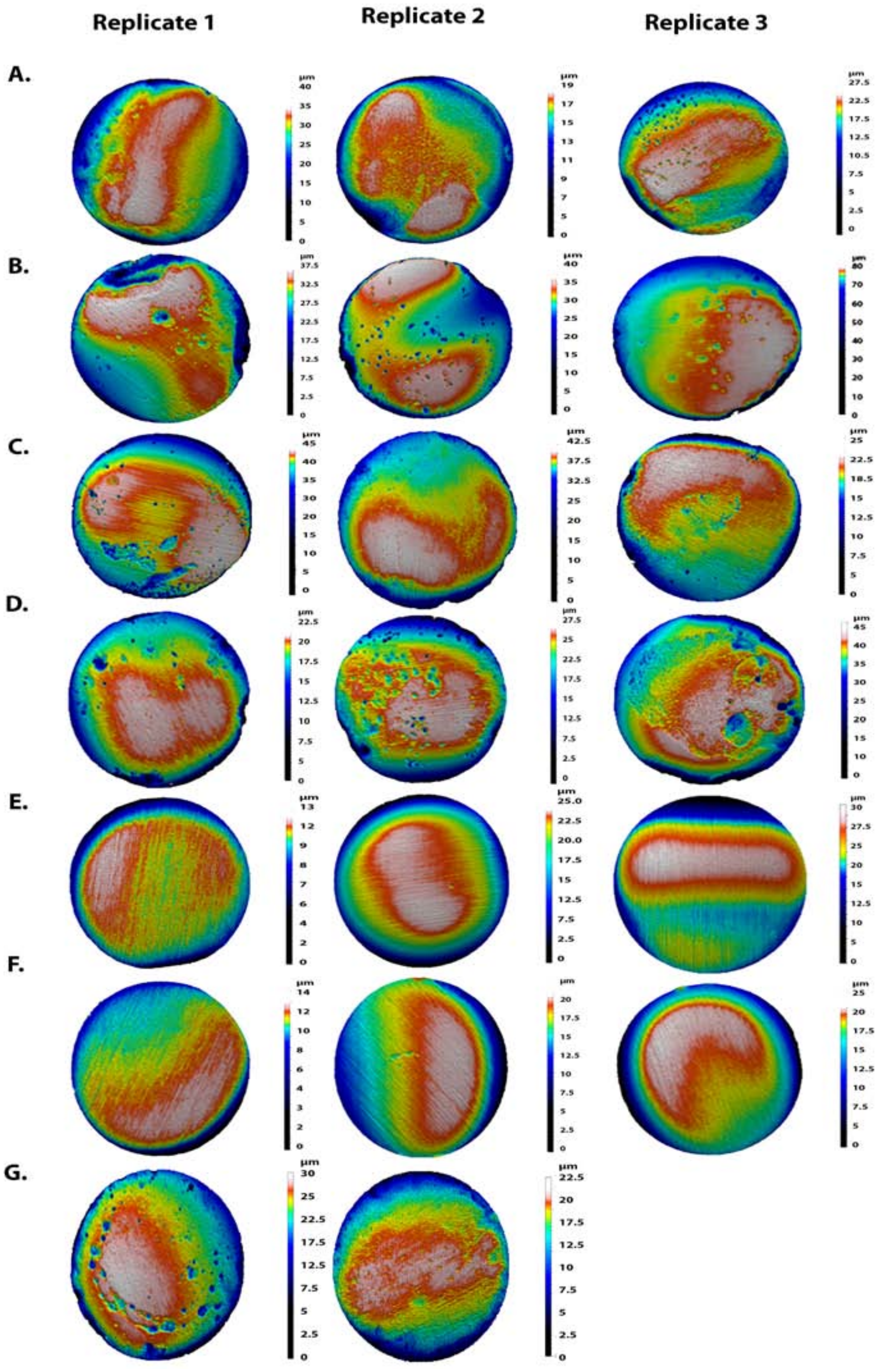

Figure S12: Surface profiles of C1020 metal coupons from experiment one exposed to pure cultures of A) *S. aciditrophicus* strain SB and co-cultures with B) *Desulfovibrio sp.* strain G11 and C) *M. hungatei* strain JF-1. Additionally, *Desulfovibrio sp*. strain G11 was cultured axenically on D) lactate, E) 138 kPa of H_2/CO_2, and F) on hydrogen from the metal surface. Also shown is an uninoculated medium control G) amended with 20 mM of crotonate. The scale bar represents surface points ranging from the highest (white) to lowest (black) for each image.

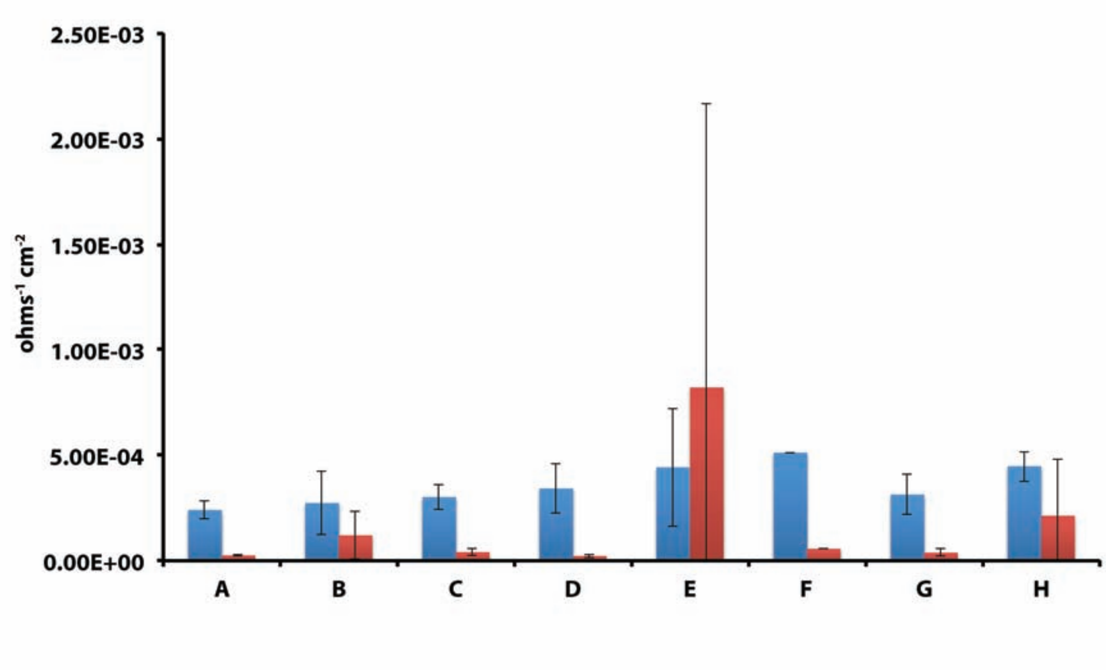

Figure S13: Instantaneous corrosion rates ($1/R_p$) for experiment two at time 0 (■) and after 28 days (■) for incubations of *S. aciditrophicus* strain SB axenically cultured on A) crotonate and B) co-cultured with *Desulfovibrio sp.* strain G11, as well as *Desulfovibrio sp.* strain G11 cultured axenically on C) lactate, D) 138 kPa of H_2/CO_2, and E) hydrogen from the metal surface. Additionally, uninoculated basal media amended with F) 20 mM of lactate G) 20 mM of crotonate, and H) 138 kPa of H_2/CO_2 or N_2/CO_2 were also monitored for corrosion.

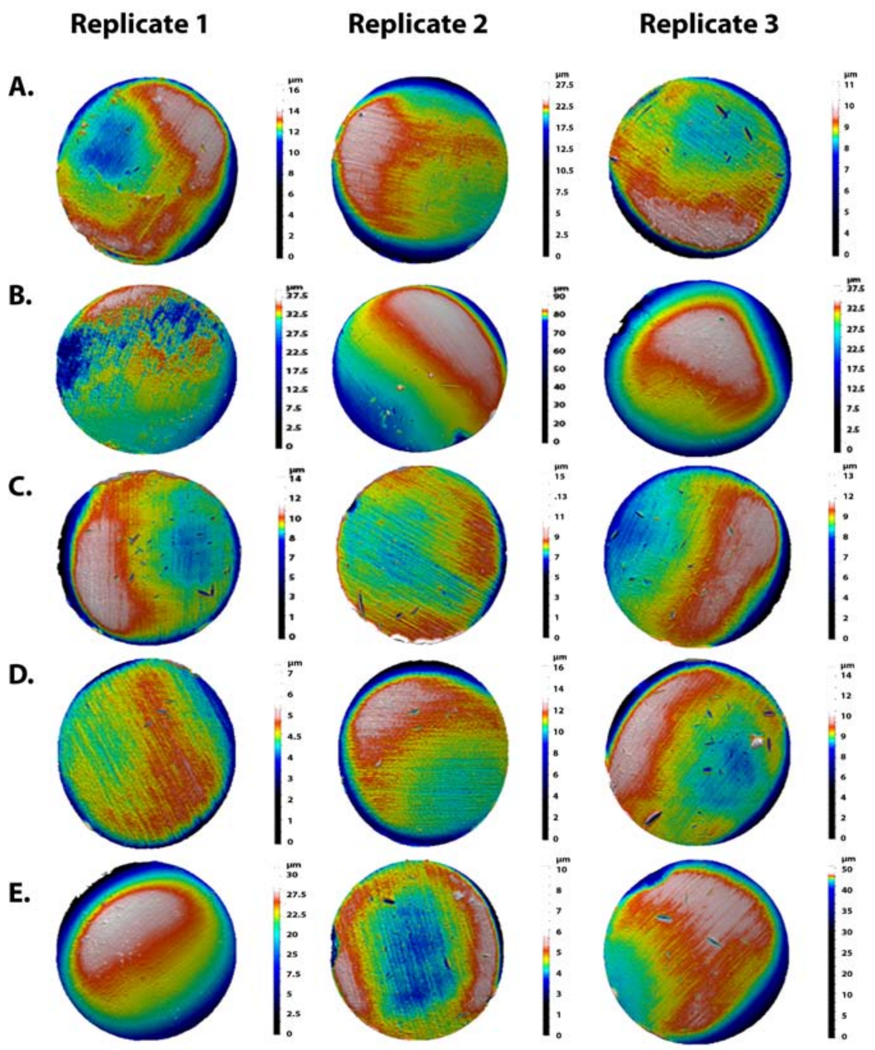

Figure S14: Surface profiles of C1020 metal coupons from experiment two exposed to pure cultures of A) *S. aciditrophicus* strain SB and co-culture incubations with B) *Desulfovibrio sp.* strain G11. Additionally, *Desulfovibrio sp*. strain G11 was axenically cultured on C) lactate, D) 138 kPa of H_2/CO_2, and E) on hydrogen from the metal surface. Replicate 3 from *Desulfovibrio sp.* strain G11 autotrophically cultured on hydrogen from the metal surface had pitting on the sides of the metal sample but the profilometer could not quantify the damage due to the curvature nature of the coupon. The scale bar represents surface points ranging from the highest (white) to lowest (black) for each image.

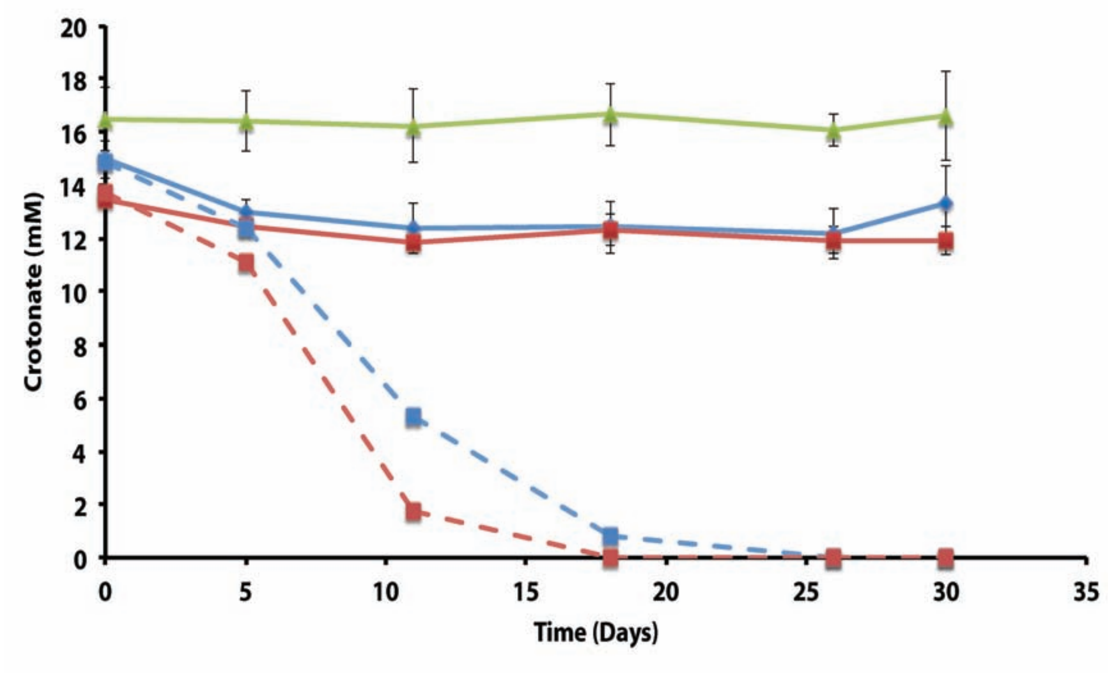

Figure S15: Crotonate depletion observed in experiment two for *S. aciditrophicus* strain SB incubations cultured axenically on crotonate (◆) and in co-culture with *Desulfovibrio sp.* strain G11 (■). No crotonate loss was observed in uninoculated basal medium controls (▲; standard deviation *n*=3). For incubations that did not contain metal samples (positive controls) crotonate was not detectable after ~18 days in axenic cultures of *S. aciditrophicus* strain SB (blue dashed line ■) and the co-culture with *Desulfovibrio sp.* strain G11 (red dashed line ■).

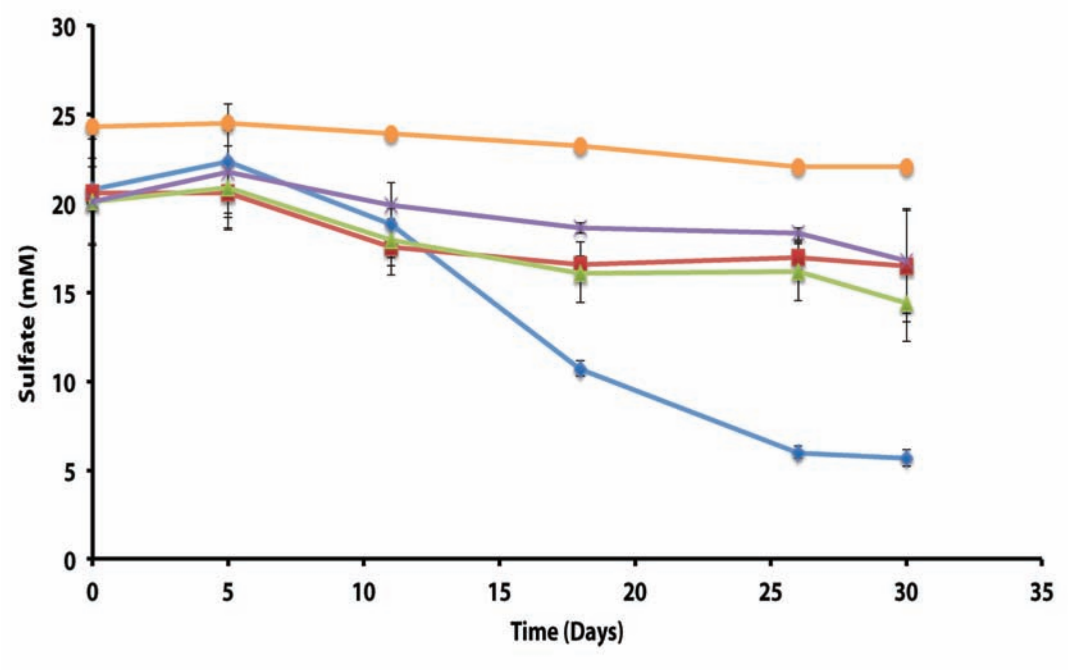

Figure S16: Sulfate reduction observed during experiment two with axenic *Desulfovibrio sp.* strain G11 cultures amended with lactate (◆), 138 kPa of H_2/CO_2 (▲), hydrogen from the metal surface (×), and in co-culture with *S. aciditrophicus* strain SB (■). No sulfate depletion was observed in uninoculated basal medium controls (●) (standard deviation *n*=3).

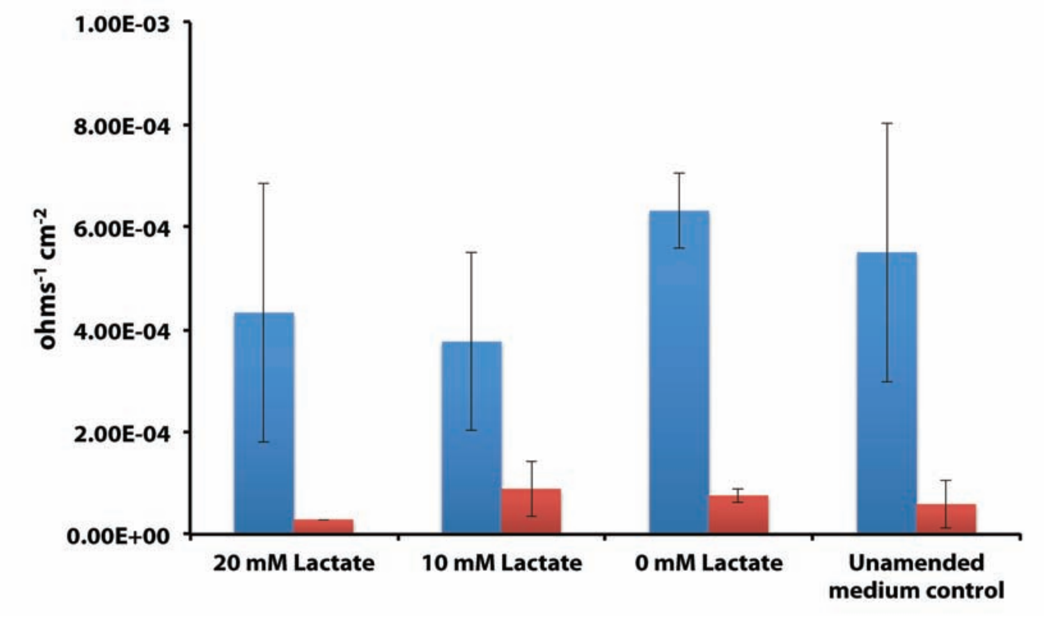

Figure S17: Instantaneous corrosion rates ($1/R_p$) at time 0 (■) and after 17 days (■) for uninoculated incubations containing exogenous amendments of lactate, acetate, and sulfide (standard deviation *n*=2).

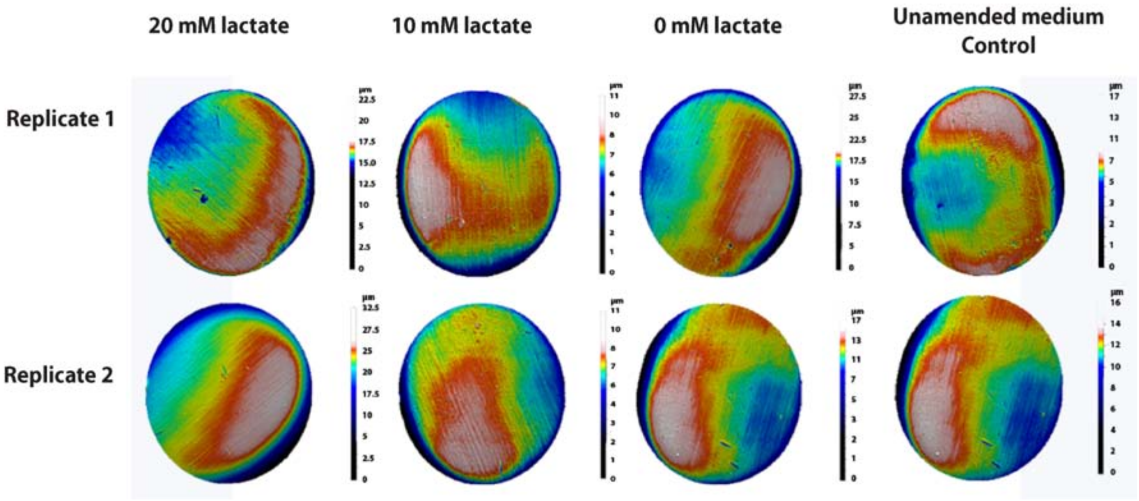

Figure S18: Surface profiles for metal samples associated with uninoculated incubations containing exogenous amendments of lactate, acetate, and sulfide. The scale bar represents surface points ranging from the highest (white) to lowest (black) for each image.

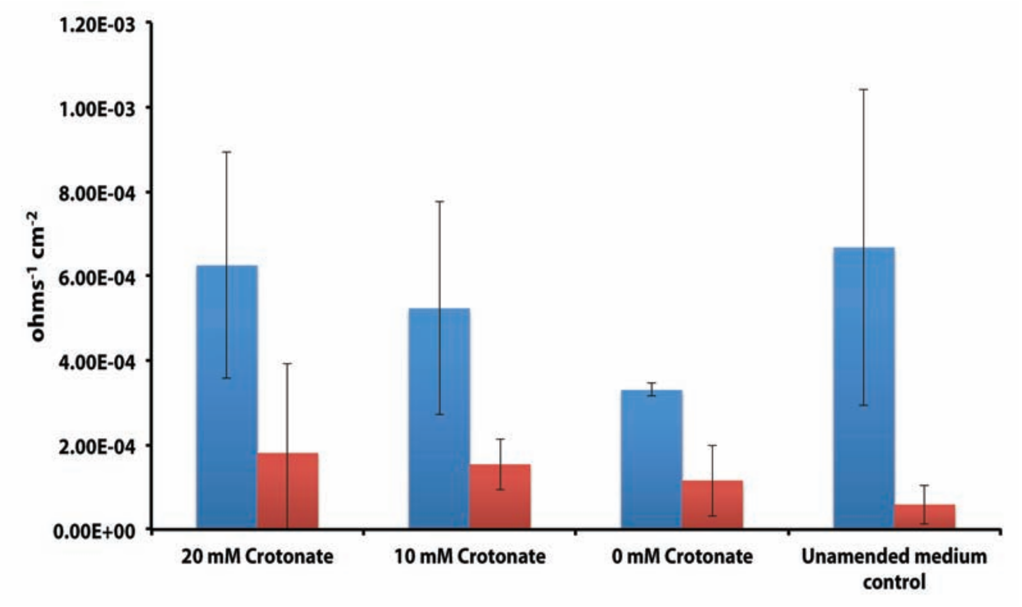

Figure S19: Instantaneous corrosion rates ($1/R_p$) rates at time 0 (■) and after 17 days (■) for uninoculated incubations containing exogenous amendments of crotonate, acetate, and sulfide (standard deviation *n*=2).

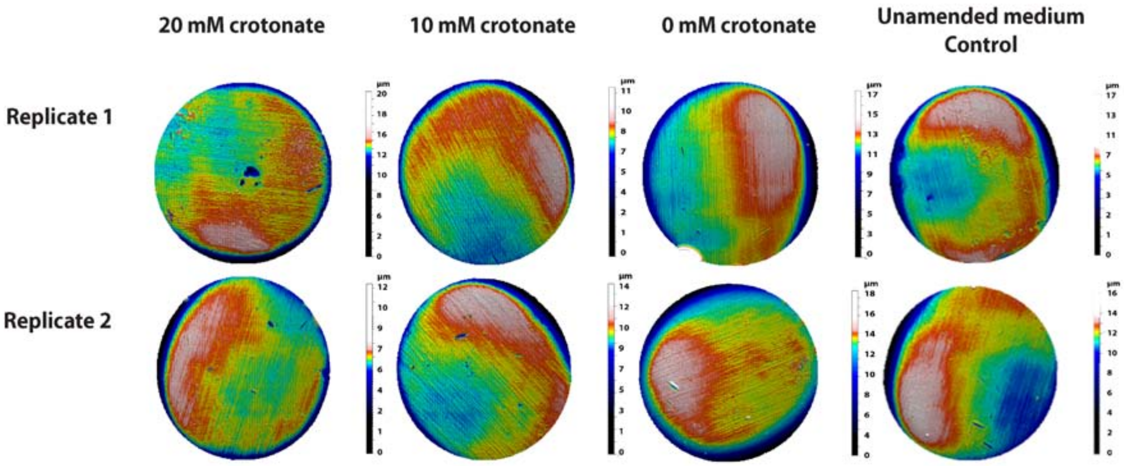

Figure S20: Surface profiles for metal samples associated with uninoculated incubations containing exogenous amendments of crotonate, acetate, and sulfide. The scale bar represents surface points ranging from the highest (white) to lowest (black) for each image.

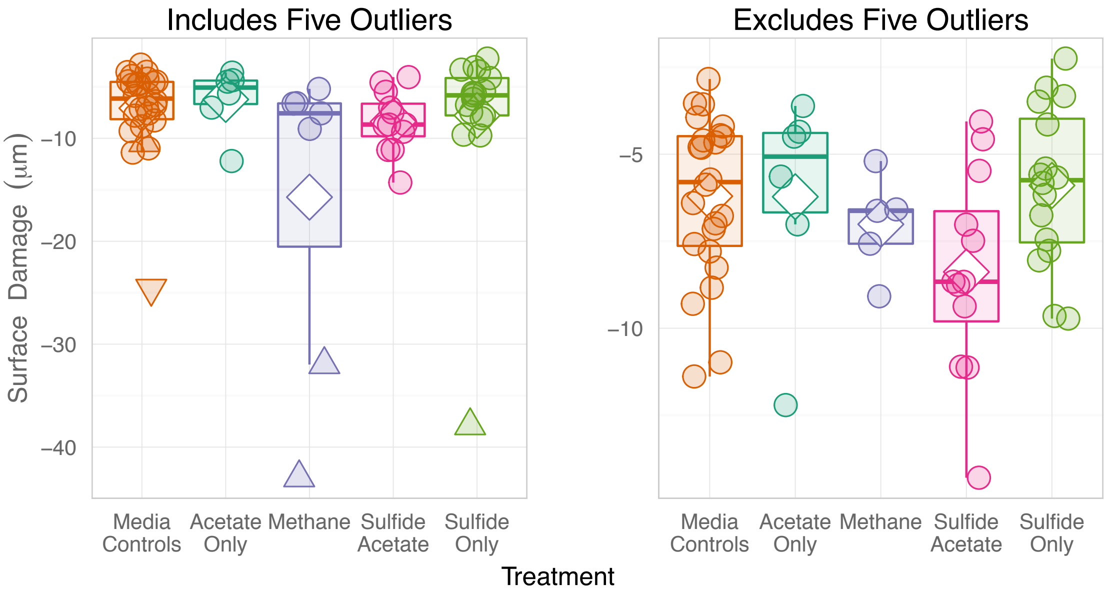

Figure S21: Box-and-whisker plots comparing coupon surface damage in the five treatment groups. The top of the box represents the 25th percentile and the bottom represents the 75th percentile of the data sets. The thick centerline is the median and the diamond is the mean. The whiskers at the top and bottom of the box represent 1.58 times the distance of the box, then it retreats toward the median until it hits an observed score. The left figure includes all data; the four coupons processed at another laboratory are marked with a triangle. An outlier coupon in the media control group is marked with an inverted triangle. The right figure (note the scale of the *y*-axis changed) excludes the five coupon outliers (below).

Coupons that were not profiled at the University of Oklahoma were considered outliers and excluded from the various statistical analyses. This analysis involved a different coupon profilometry protocol (Figure S8), and the resulting histograms were not comparable (triangles). Additionally, one coupon from an uninoculated medium control was excluded. This coupon (inverted triangle) had a higher mean depth distribution (~25 µm) compared to the other media control coupons. This increased depth distribution was attributed to a defect area on the surface of the coupon, though no pits matching the experimental parameters were identified (Table 4). Considering the other 24 media control coupons had a mean depth of ~10 µm, this coupon was excluded from the various statistical analyses.
